# Supplementary material for: Characterization of Colorants Formed by Non-Enzymatic Browning Reactions of Hydroxycinnamic Acid Derivatives
Source: Molecules. 2022 Nov 4;27(21):7564. doi: 10.3390/molecules27217564 (PMC9658777; doi:10.3390/molecules27217564)
Supplement: Supplementary file 1 [file molecules-27-07564-s001.zip › molecules-2008131-supplementary.pdf]

Supplementary Material

Characterization of Colorants Formed by Non-Enzymatic Browning Reactions of Hydroxycinnamic Acid Derivatives

Leon Valentin Bork *, Sascha Rohn and Clemens Kanzler *

Institute of Food Technology and Food Chemistry, Department of Food Chemistry and Analysis, Technische Universität Berlin, Gustav-Meyer-Allee 25, 13355 Berlin, Germany
* Correspondence: l.bork@tu-berlin.de (L.V.B.); clemens.kanzler@tu-berlin.de (C.K.); Tel.: +49-30-314-72404 (C.K.)

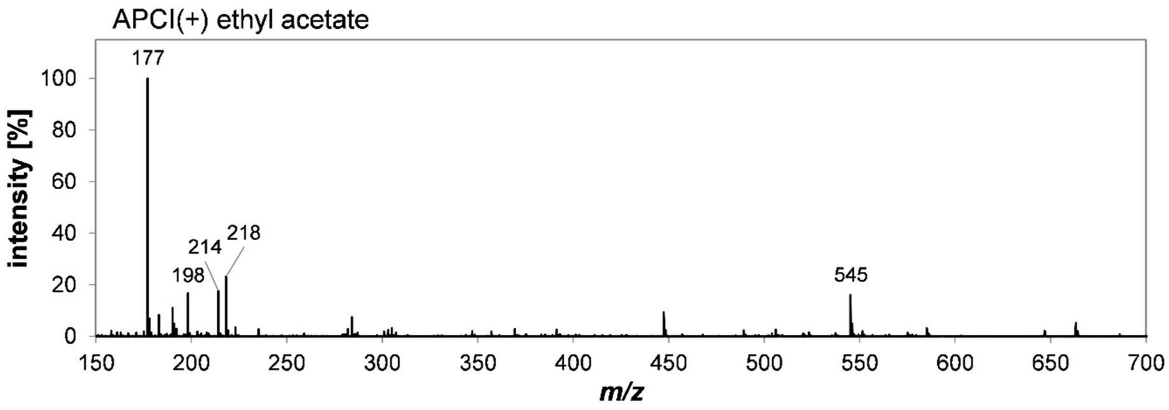

Figure S-1. HRMS spectrum of ethyl acetate with commonly found solvent signals at *m*/*z* 177, 198, *m*/*z* 214, *m*/*z* 218, and *m*/*z* 545.

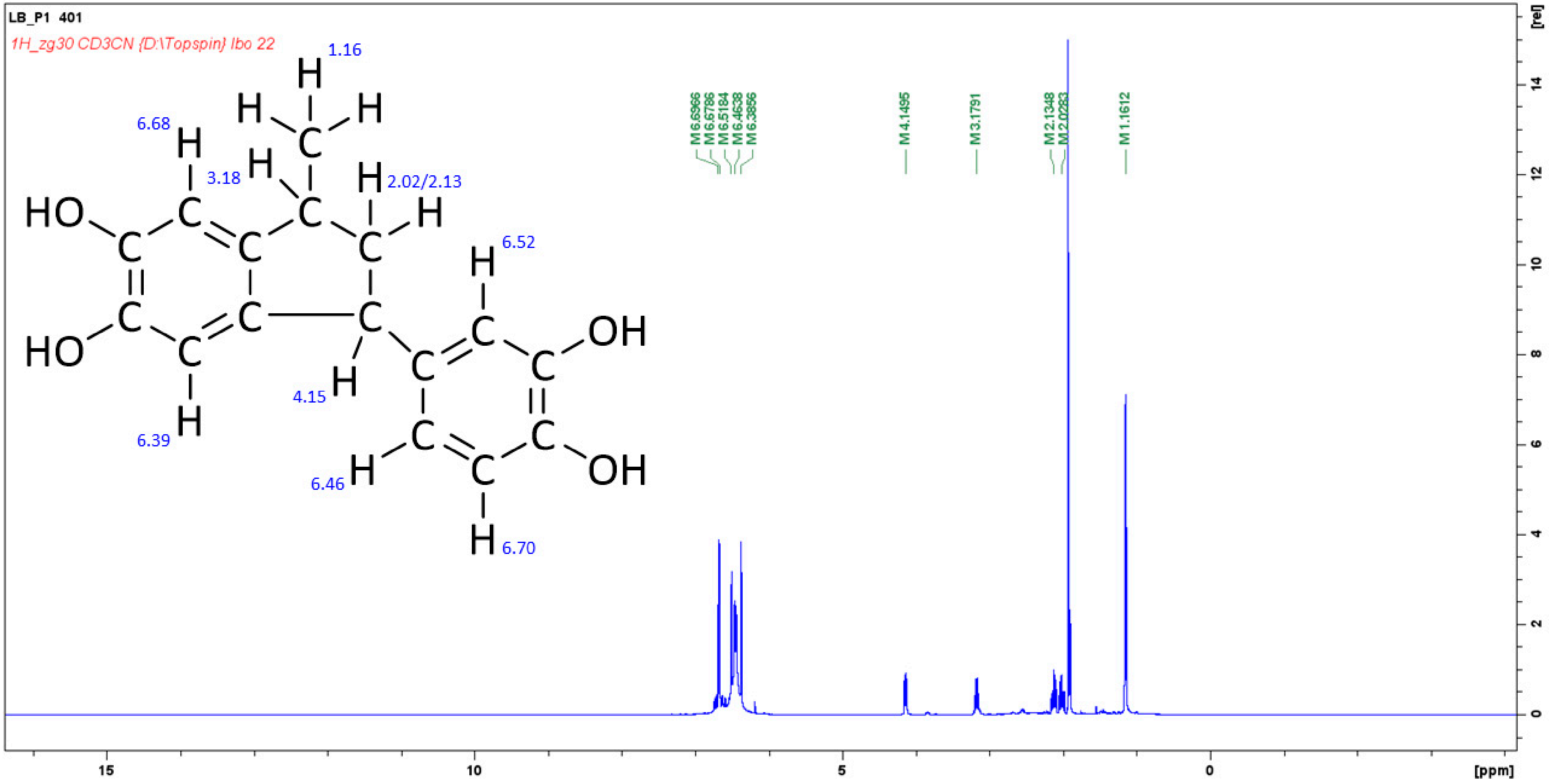

Figure S-2. 1H-NMR spectrum of 1-(3,4-dihydroxyphenyl)-3-methyl-2,3dihydro-1H-indene-5,6-diol (P1).

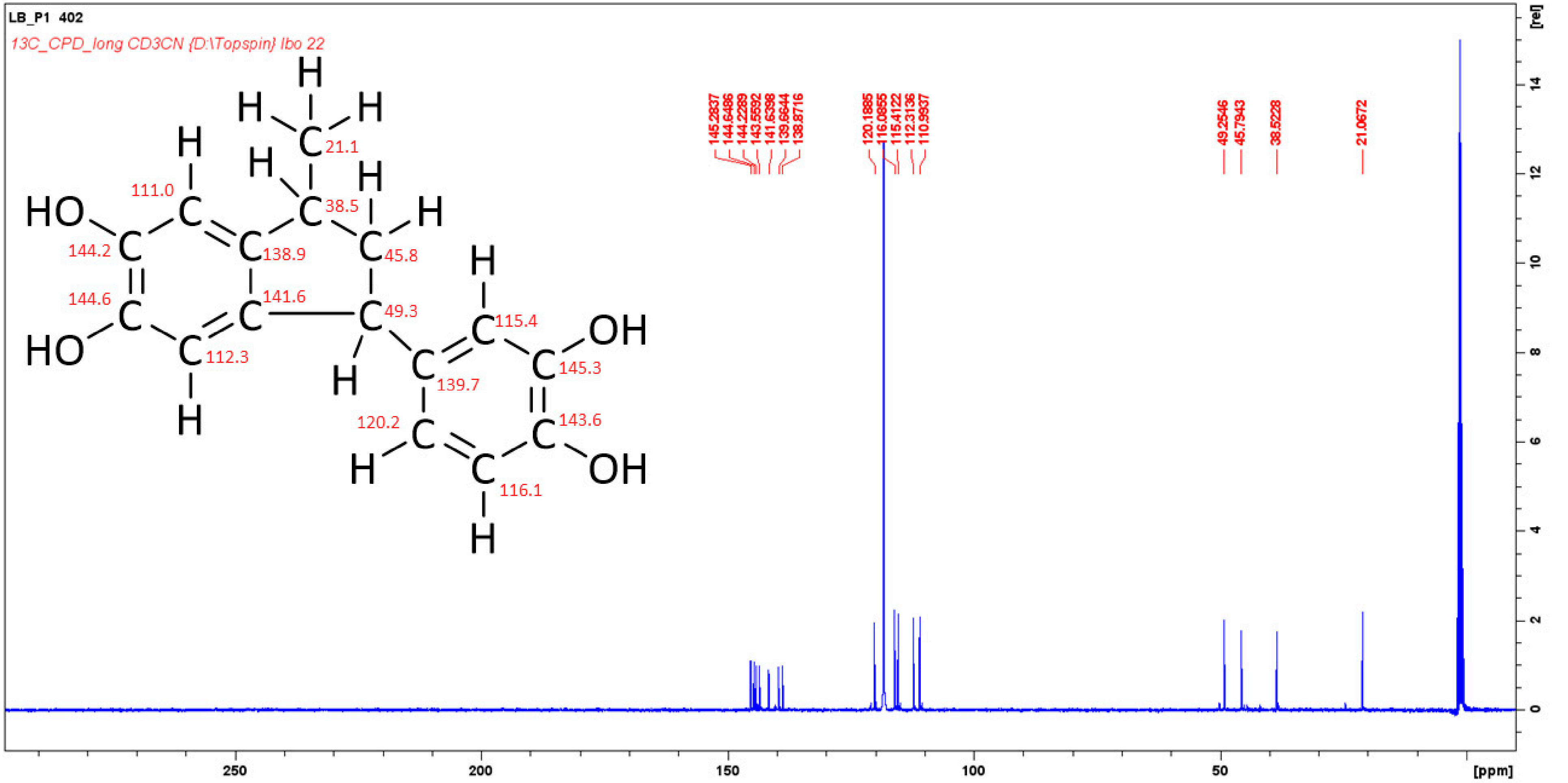

Figure S-3. ^{13}C-NMR spectrum of 1-(3,4-dihydroxyphenyl)-3-methyl-2,3dihydro-1*H*-indene-5,6-diol (P1).

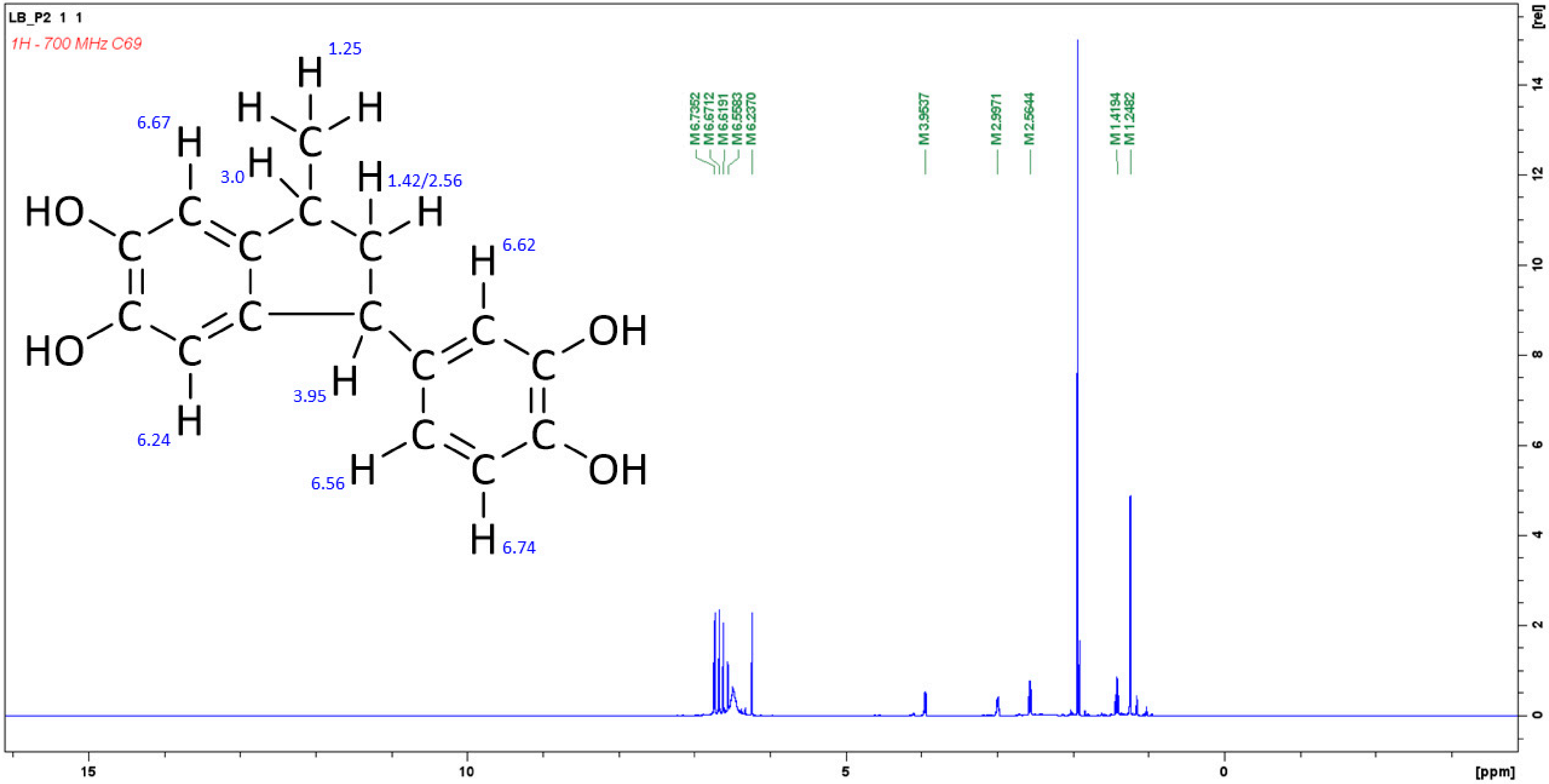

Figure S-4. ^{1}H-NMR spectrum of 1-(3,4-dihydroxyphenyl)-3-methyl-2,3dihydro-1*H*-indene-5,6-diol (P2).

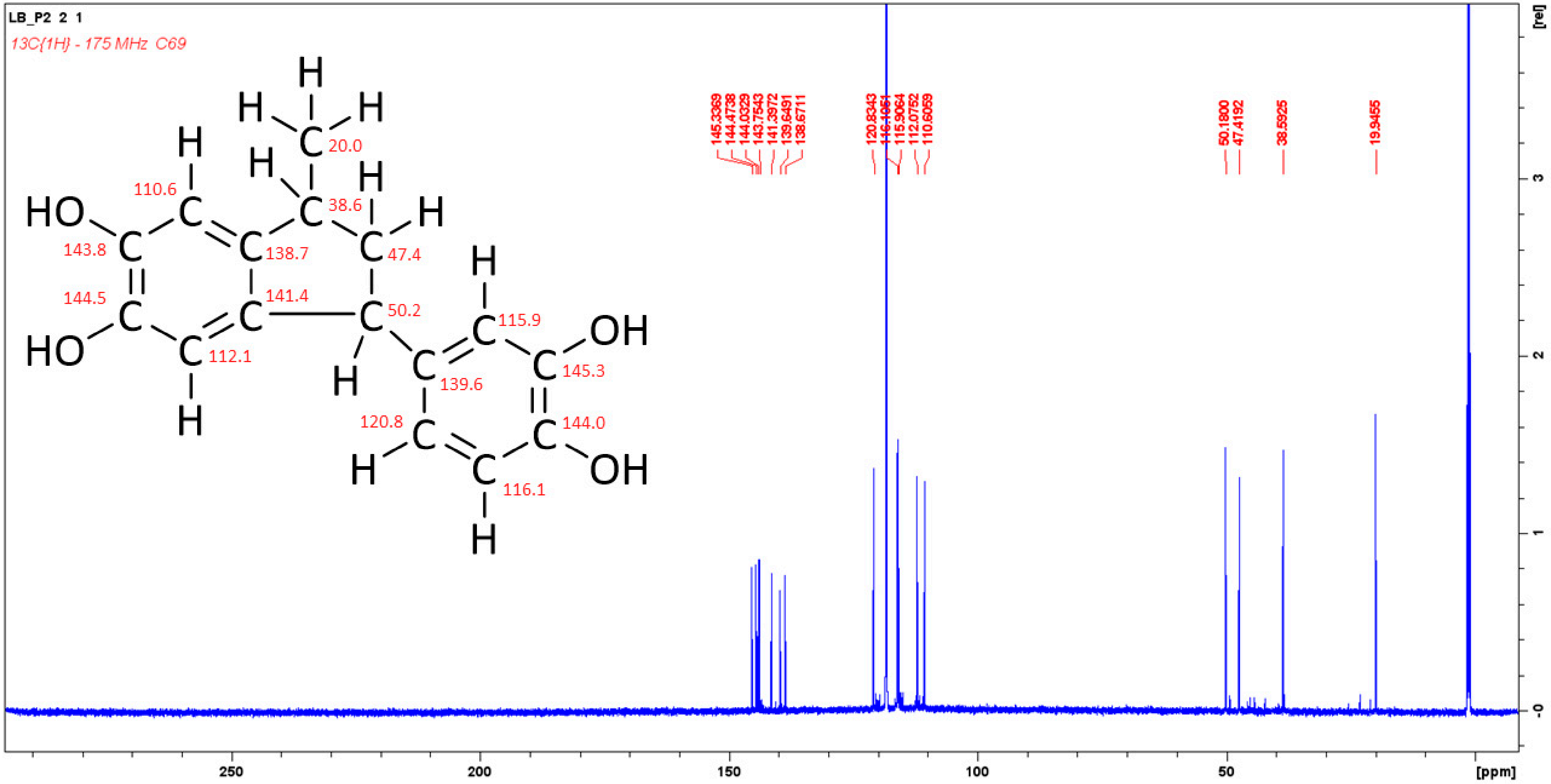

Figure S-5. ^{13}C-NMR spectrum of 1-(3,4-dihydroxyphenyl)-3-methyl-2,3dihydro-1*H*-indene-5,6-diol (P2).

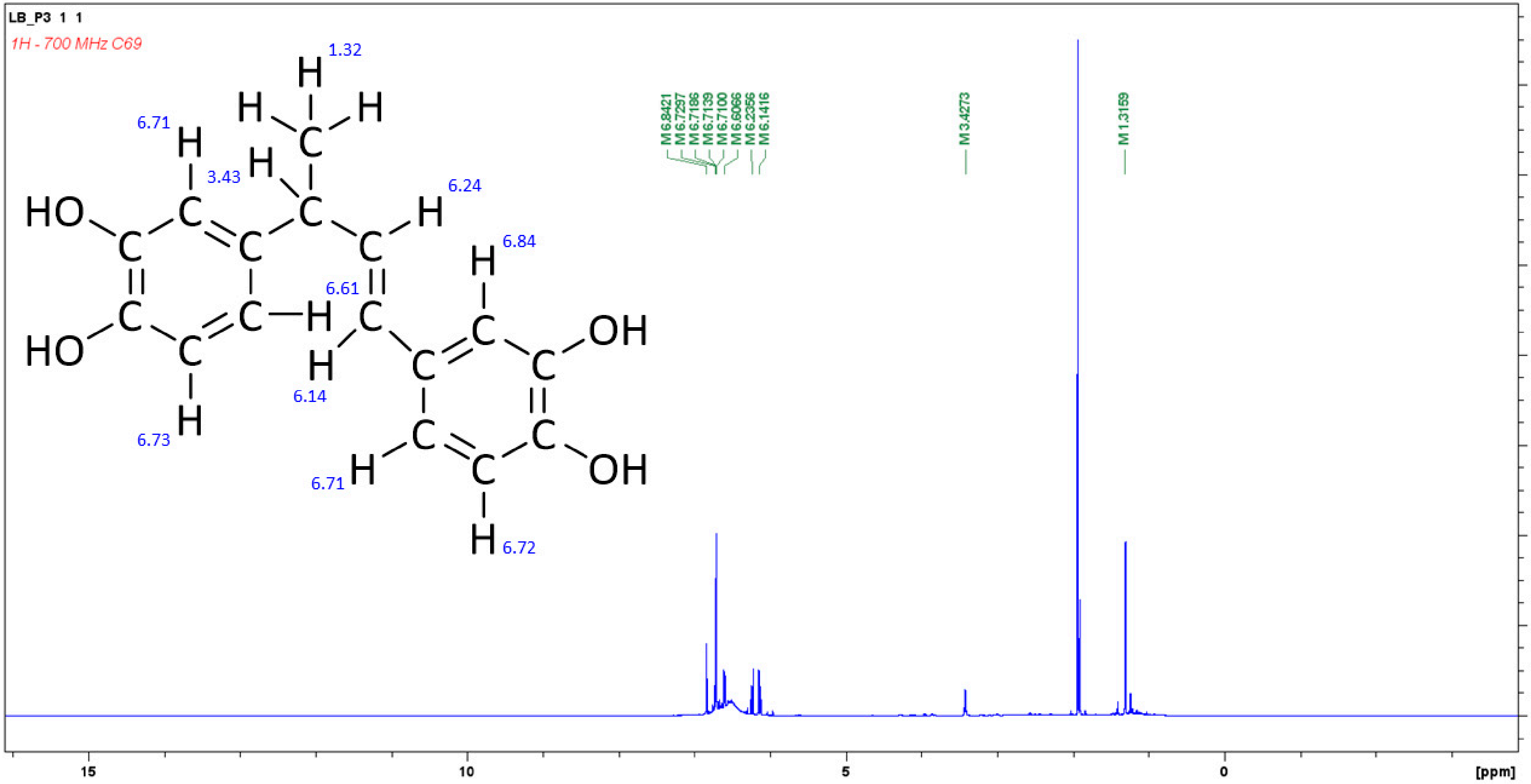

Figure S-6. ^{1}H-NMR spectrum of (*E*)-4,4′-(but-1-ene-1,3-diyl)bis(benzene-1,2-diol) (P3).

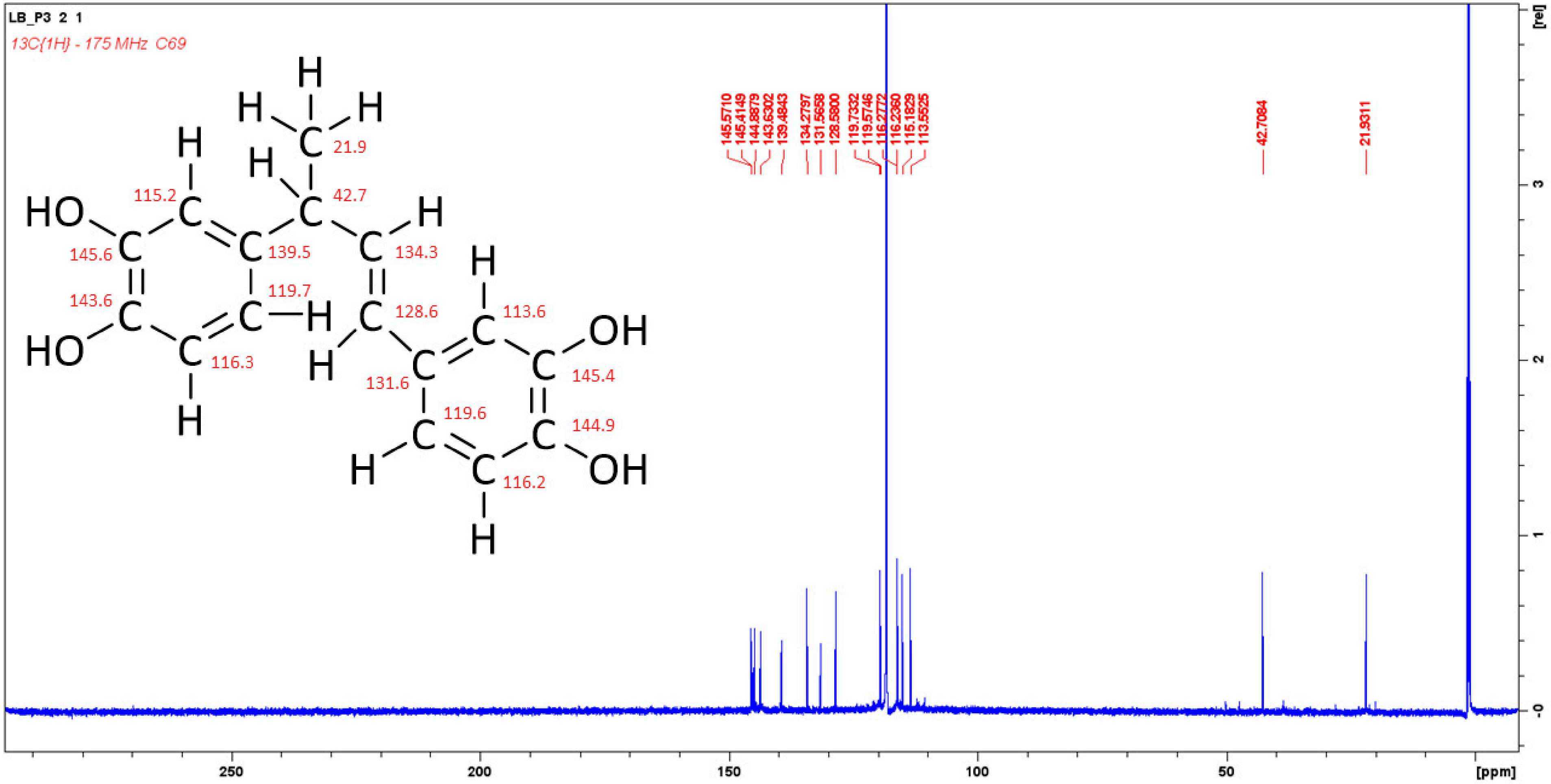

Figure S-7. ^{13}C-NMR spectrum of (*E*)-4,4′-(but-1-ene-1,3-diyl)bis(benzene-1,2-diol) (P3).

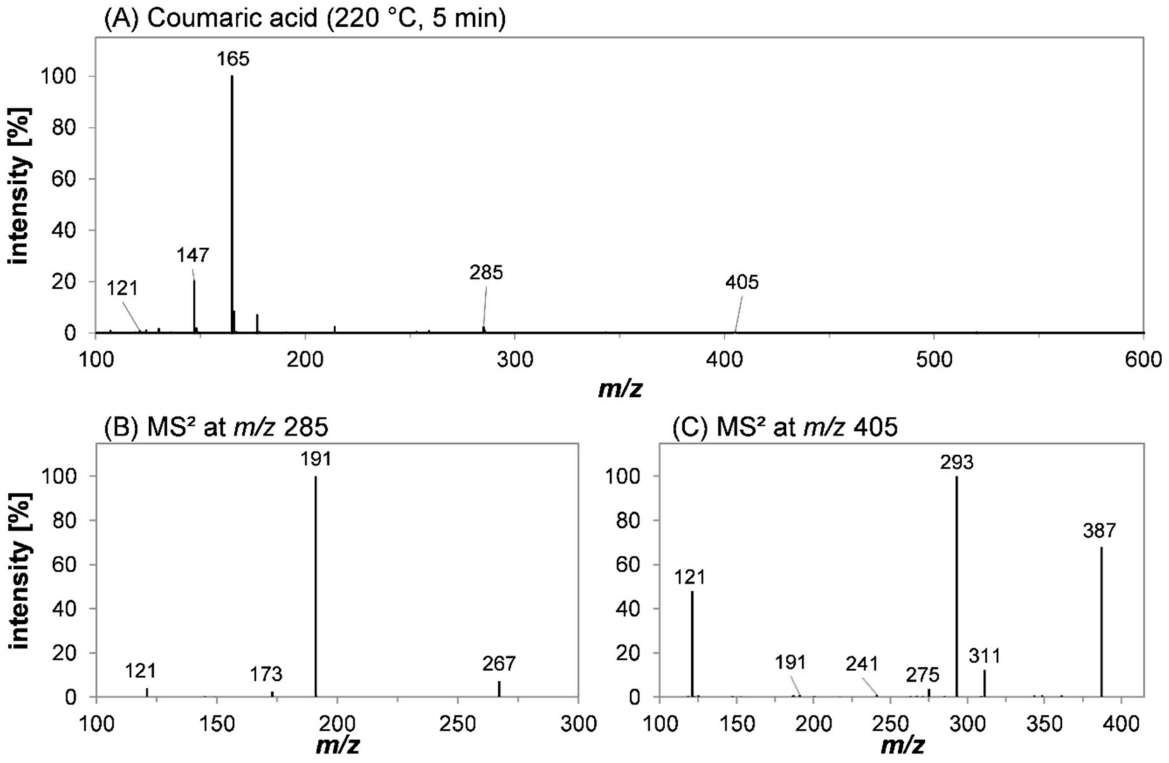

Figure S-8. HRMS analysis of heat-treated coumaric acid (CS) (220 °C. 5 min) measured after APCI in positive ion mode. (A) Scan spectrum and fragmentation spectra after CID of (B) *m/z* 285. and (C) *m/z* 405.

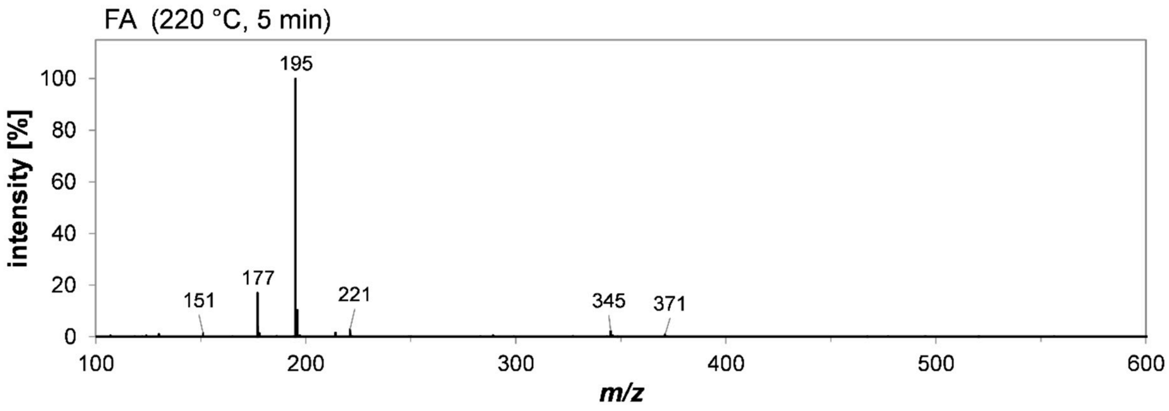

Figure S-9. HRMS scan spectrum of heat-treated ferulic acid (FA) (220 °C. 5 min) measured after APCI in positive ion mode.

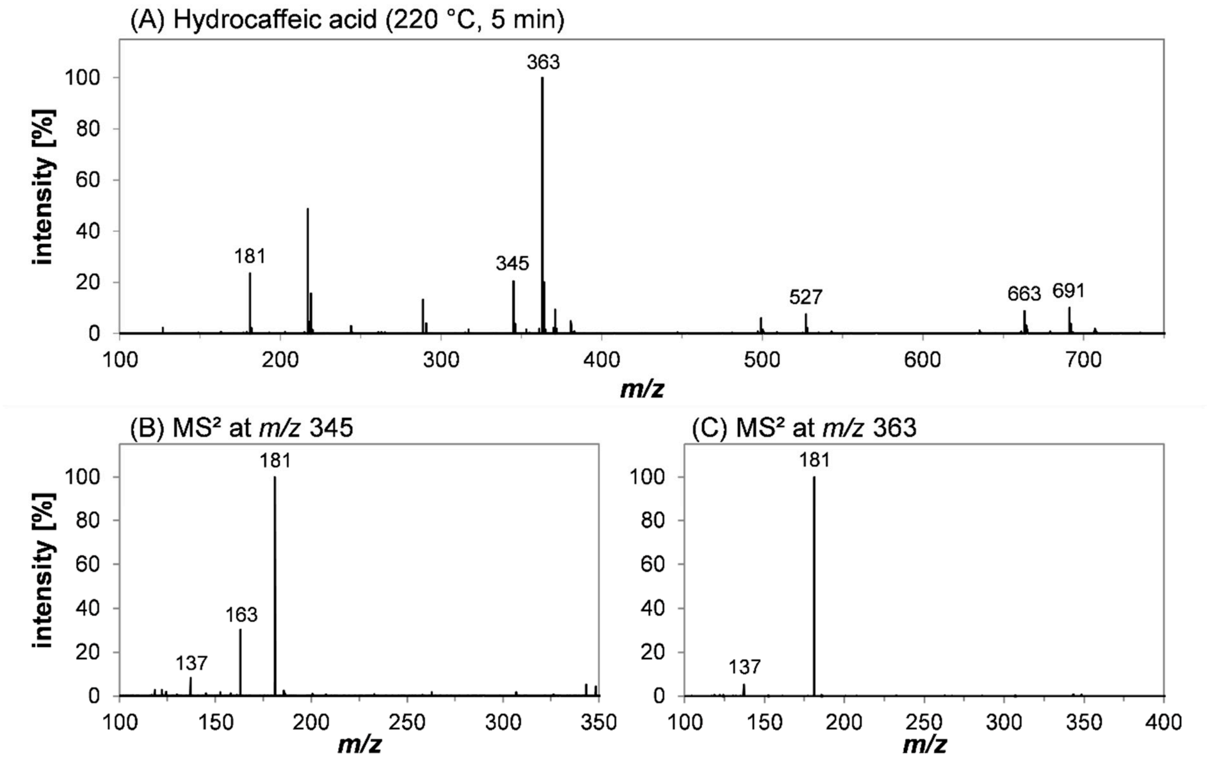

Figure S-10. HRMS analysis of heat-treated hydrocaffeic acid (HC) (220 °C. 5 min) measured after ESI in negative ion mode. (A) Scan spectrum and fragmentation spectra after CID of (B) *m/z* 345. and (C) *m/z* 363.

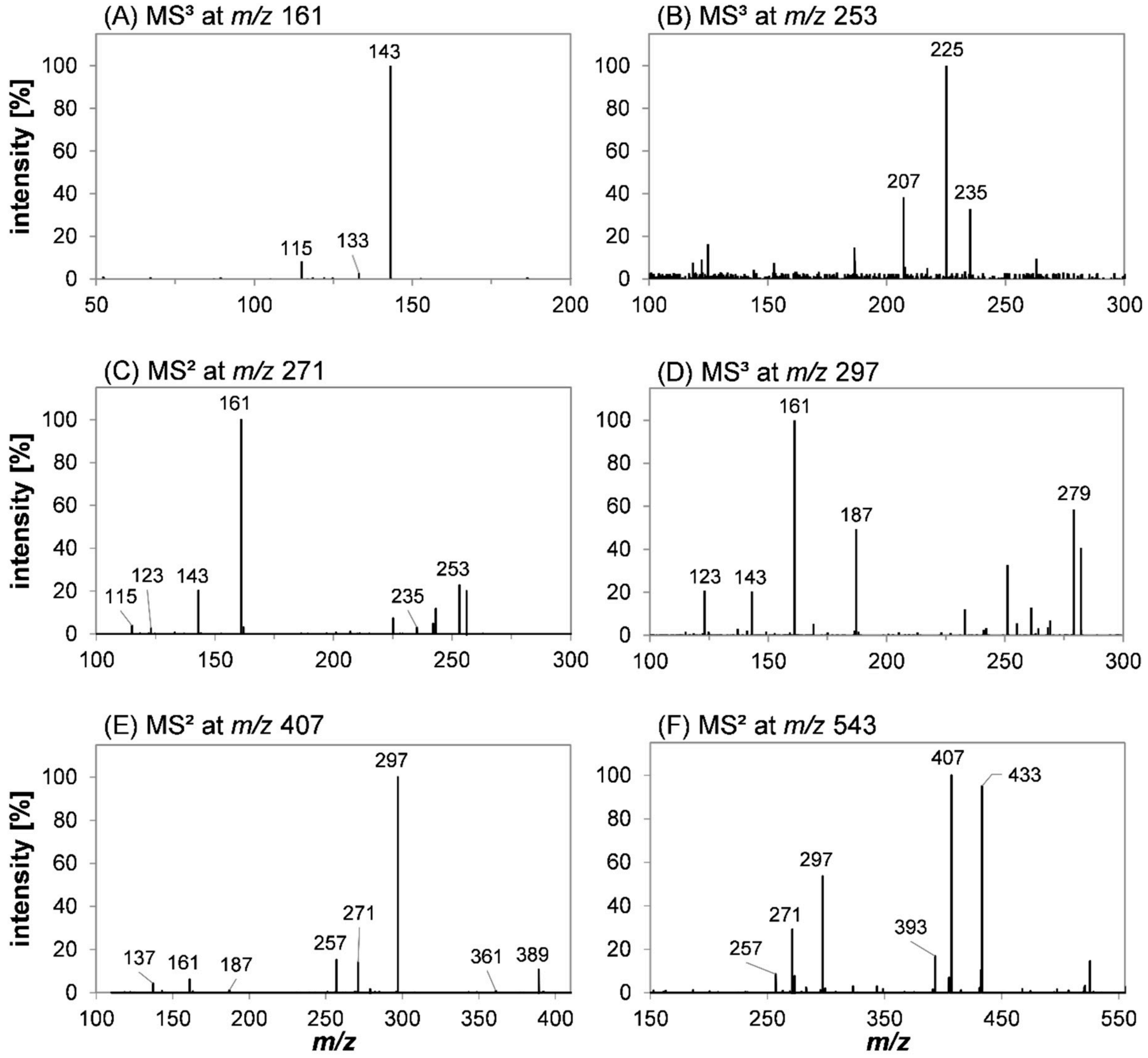

Figure S-11. Fragmentation spectra of (A) *m/z* 161, (B) *m/z* 253, (C) *m/z* 271, (D) *m/z* 297, (E) *m/z* 407, and (F) *m/z* 543 recorded *via* HRMS^n of caffeic acid (CA) after heat-treatment (220 °C. 5 min) using APCI in positive ion mode.

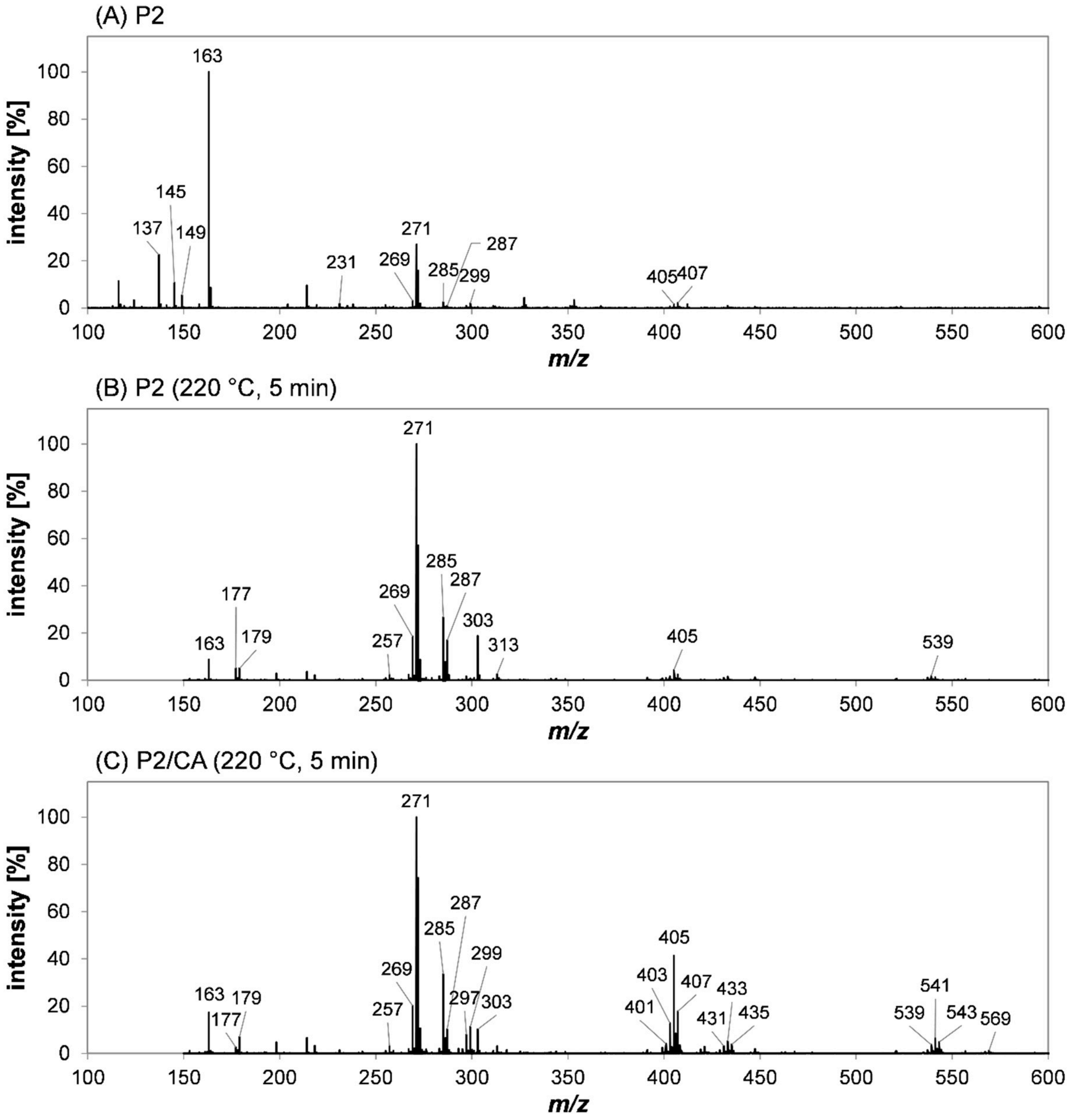

Figure S-12. Mass spectra of (A) P2, (B) P2 at 220 °C for 5 min, and (C) P2/CA at 220 °C for 5 min. Mass spectra were modified by manually setting the intensity of significant signals to zero which were resulting from ionization of the solvent (e.g. ethyl acetate dimer at *m/z* 177, *m/z* 214, *m/z* 545). A scan spectrum of the solvent can be found in the supplementary material (Figure S-1).

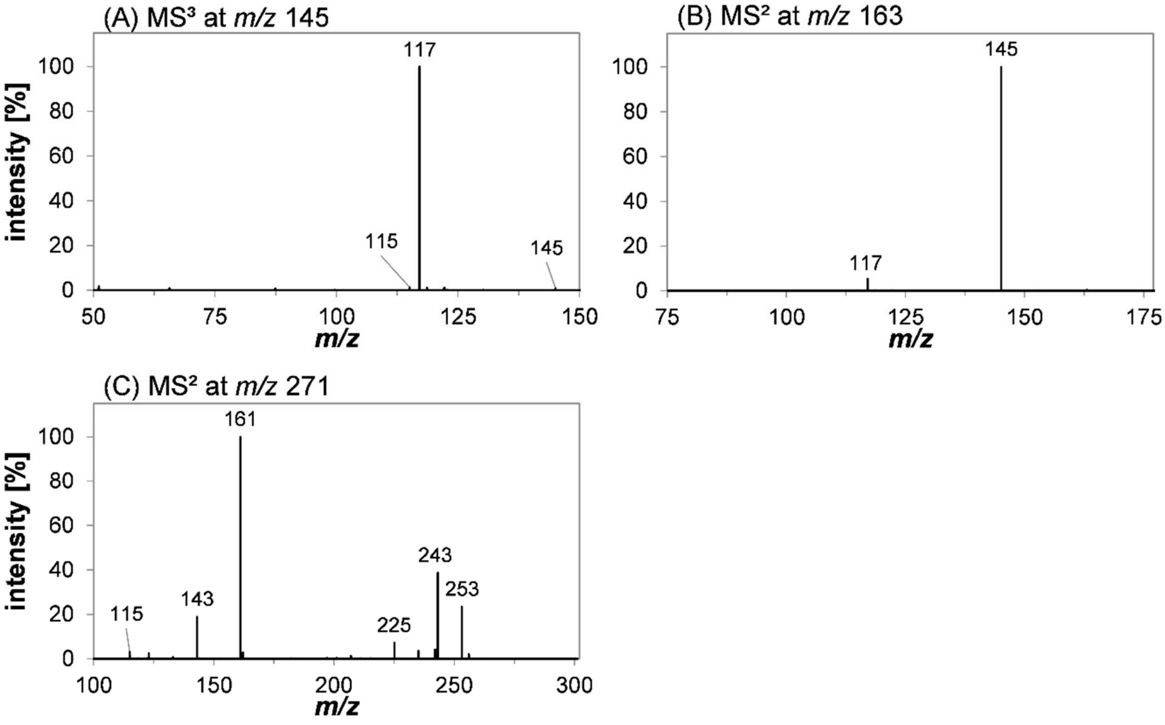

Figure S-13. Fragmentation spectra of (A) *m*/*z* 145, (B) *m*/*z* 163, and (C) *m*/*z* 271 recorded *via* HRMS^n of P1 using APCI in positive ion mode.

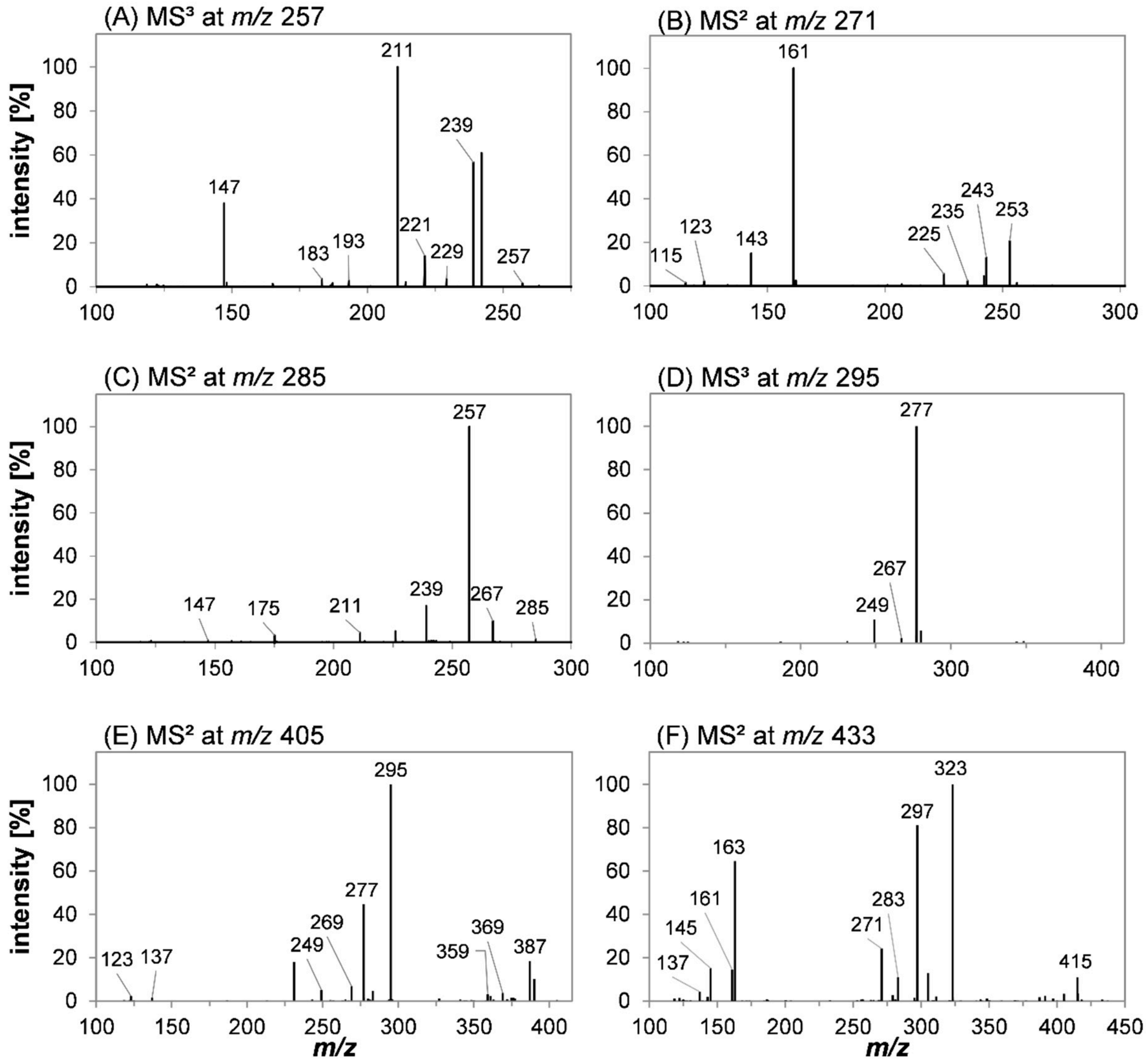

Figure S-14. Fragmentation spectra of (A) *m/z* 257, (B) *m/z* 271, (C) *m/z* 285, (D) *m/z* 295, (E) *m/z* 405, and (F) *m/z* 433 recorded *via* $HRMS^n$ of P1 after heat-treatment (220 °C. 5 min) using APCI in positive ion mode.

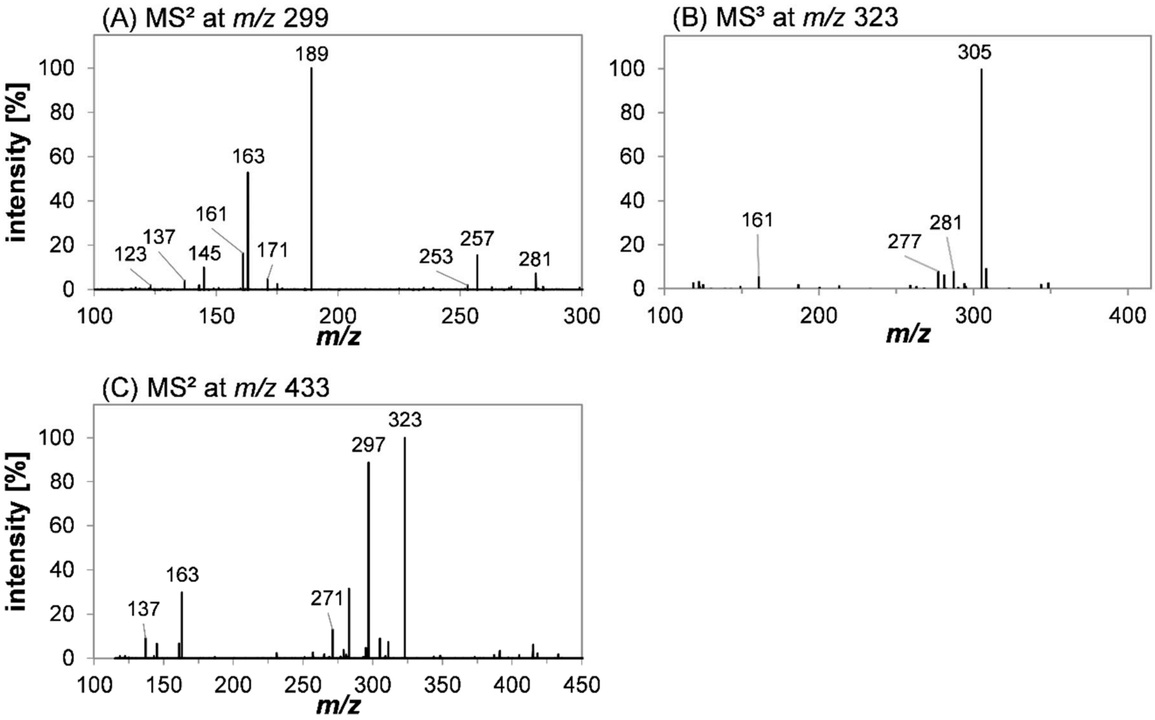

Figure S-15. Fragmentation spectra of (A) *m/z* 299, and (B) *m/z* 323, and (C) *m/z* 433 recorded *via* HRMS^n of P1/CA after heat-treatment (220 °C. 5 min) using APCI in positive ion mode.

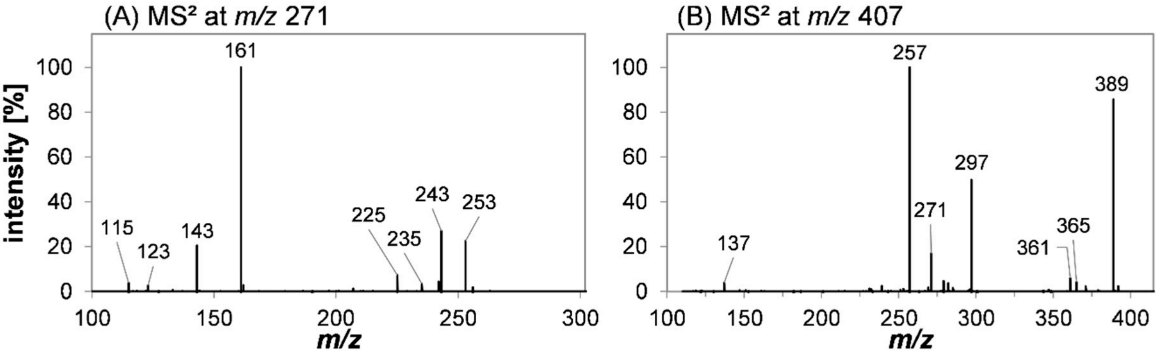

Figure S-16. Fragmentation spectra of (A) *m/z* 271, and (B) *m/z* 407 recorded *via* tandem HRMS of P2 using APCI in positive ion mode.

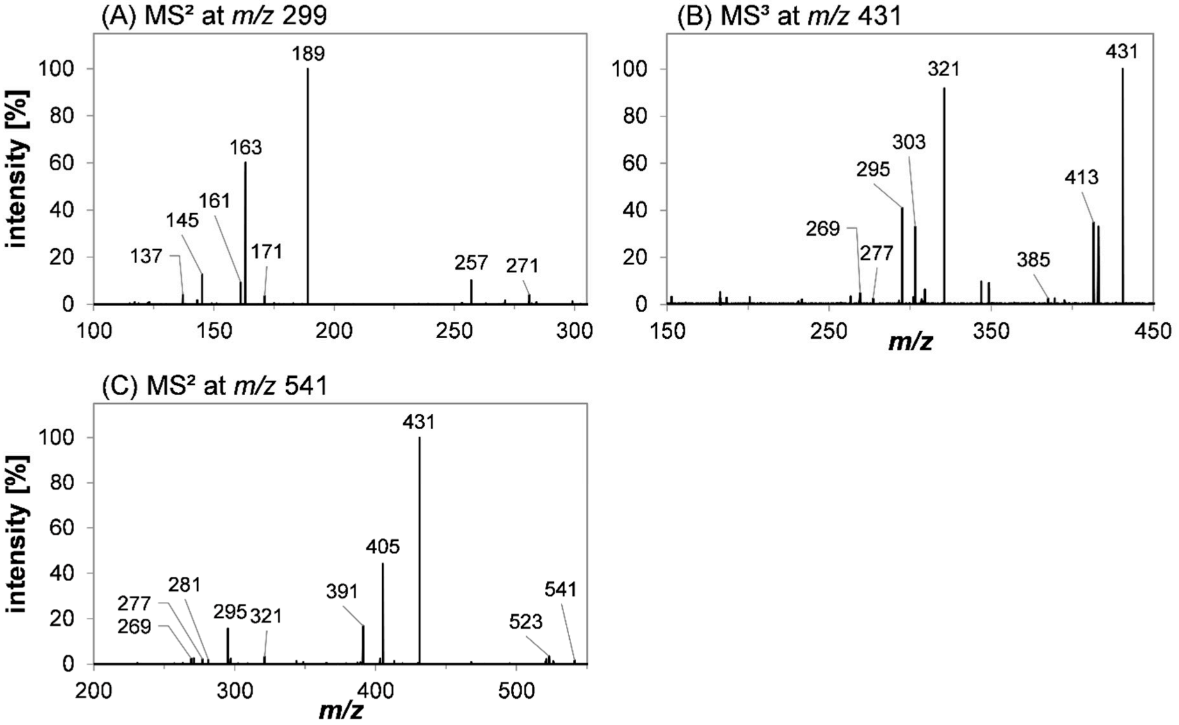

Figure S-17. Fragmentation spectra of (A) *m*/*z* 299, and (B) *m*/*z* 431, and (C) *m*/*z* 541 recorded *via* HRMS^n of P2/CA after heat-treatment (220 °C. 5 min) using APCI in positive ion mode.

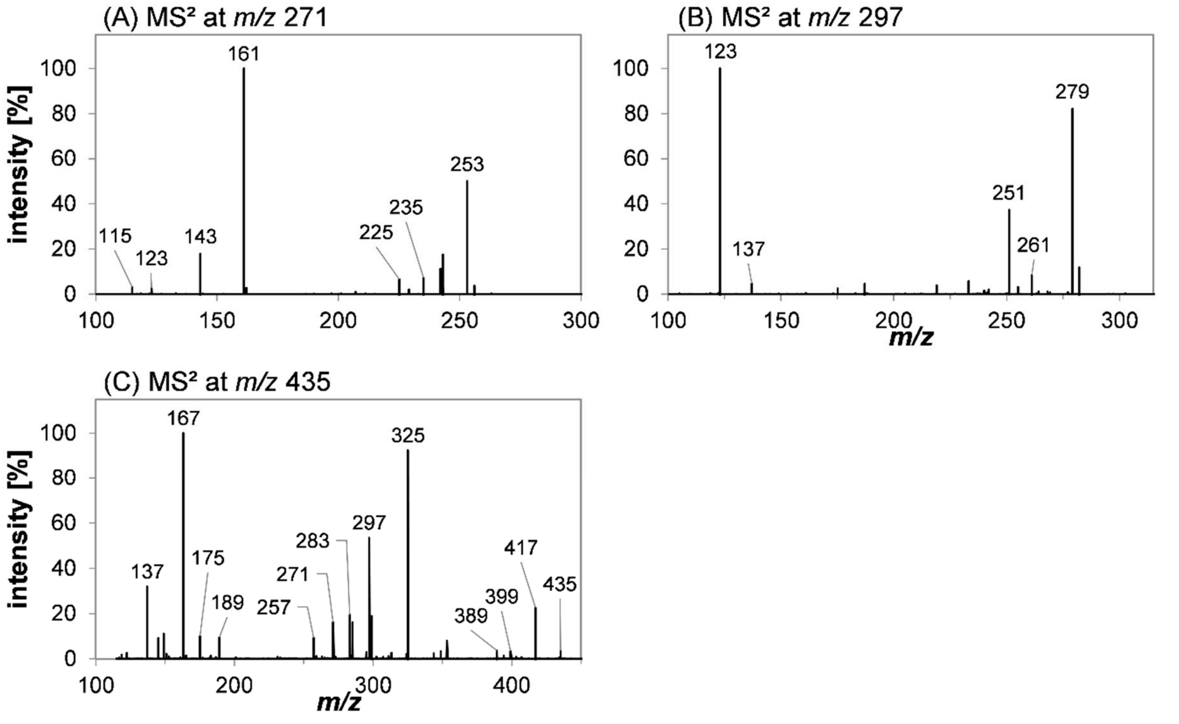

Figure S-18. Fragmentation spectra of (A) *m*/*z* 271, and (B) *m*/*z* 297, and (C) *m*/*z* 435 recorded *via* tandem HRMS of P3 using APCI in positive ion mode.

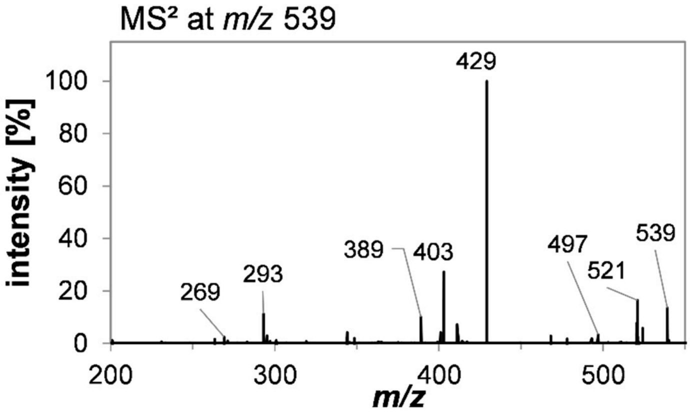

Figure S-19. Fragmentation spectrum of *m*/*z* 539 recorded *via* tandem HRMS of P3/CA after heat-treatment (220 °C. 5 min) using APCI in positive ion mode.

Table S-1. Assignment of signals obtained by HRMS analysis of caffeic acid (CA) at 220 °C for 5 min measured using an APCI-orbitrap-MS instrument in positive ion mode.

	structure assignment				mol.	*m/z*		rel. error	rel.
CA	CO_2	$C_6H_6O_2$	H_2O	H_2	formula	exp.	theo.	[ppm]	intensity
1	–1	0	0	0	$C_8H_9O_2H^+$	137.0595	137.0597	–1.5	100.0
2	–2	–1	0	0	$C_{10}H_{10}O_2H^+$	163.0751	163.0754	–1.8	37.2
2	–2	0	0	–2	$C_{16}H_{12}O_4H^+$	269.0807	269.0808	–0.4	14.0
2	–2	0	0	–1	$C_{16}H_{14}O_4H^+$	271.0963	271.0965	–0.7	77.6
3	–3	0	0	–2	$C_{24}H_{20}O_6H^+$	405.1328	405.1332	–1.0	34.8
3	–3	0	0	–1	$C_{24}H_{22}O_6H^+$	407.1484	407.1489	–1.2	38.9
4	–4	0	0	–2	$C_{32}H_{28}O_8H^+$	541.1852	541.1857	–0.9	5.0
4	–4	0	0	–1	$C_{32}H_{30}O_8H^+$	543.2008	543.2013	–0.9	8.0

Table S-2. Assignment of signals obtained by HRMS analysis of *p*-coumaric acid (CS) at 220 °C for 5 min measured using an APCI-orbitrap-MS instrument in positive ion mode.

	structure assignment				mol.	*m/z*		rel. error	rel.
CS	CO_2	$C_6H_6O_2$	H_2O	H_2	formula	exp.	theo.	[ppm]	intensity
1	–1	0	0	0	$C_8H_8OH^+$	121.0646	121.0648	–1.6	0.9
1	0	0	–1	0	$C_9H_6O_2H^+$	147.0439	147.0441	–1.1	20.4
1	0	0	0	0	$C_9H_8O_3H^+$	165.0543	165.0546	–1.9	100.0
2	–1	0	0	0	$C_{17}H_{16}O_4H^+$	285.1119	285.1121	–0.8	2.4
3	–2	0	0	0	$C_{25}H_{24}O_5H^+$	405.1693	405.1697	–0.9	0.4

Table S-3. Assignment of signals obtained by HRMS analysis of ferulic acid (FA) at 220 °C for 5 min measured using an APCI-orbitrap-MS instrument in positive ion mode.

	structure assignment			mol.	*m*/*z*		rel. error	rel.
**FA**	**CO_2**	**$C_7H_8O_2$**	**H_2O**	**formula**	**exp.**	**theo.**	**[ppm]**	**intensity**
1	–1	0	0	$C_9H_{10}O_2H^+$	151.0753	151.0754	–0.4	1.5
1	0	0	–1	$C_{10}H_8O_3H^+$	177.0544	177.0546	–1.2	17.0
1	0	0	0	$C_{10}H_{10}O_4H^+$	195.0650	195.0652	–1.0	100.0
2	–1	–1	0	$C_{12}H_{12}O_4H^+$	221.0807	221.0808	–0.6	2.9
2	0	–1	0	$C_{19}H_{20}O_6H^+$	345.1332	345.1333	–0.2	2.3
2	0	0	–1	$C_{20}H_{18}O_7H^+$	371.1125	371.1125	–0.1	1.1

Table S-4. Assignment of signals obtained by HRMS analysis of hydrocaffeic acid (HC) at 220 °C for 5 min measured using an ESI-orbitrap-MS instrument in negative ion mode.

	structure assignment			mol.	*m*/*z*		rel. error	rel.
**HC**	**CO_2**	**H_2O**	**H_2**	**formula**	**exp.**	**theo.**	**[ppm]**	**intensity**
1	0	0	0	$C_9H_9O_4^-$	181.0507	181.0506	0.4	23.5
2	0	–1	0	$C_{18}H_{17}O_7^-$	345.0979	345.0969	3.0	20.4
2	0	0	0	$C_{18}H_{19}O_8^-$	363.1084	363.1085	–0.5	100.0
3	0	–1	0	$C_{27}H_{27}O_{11}^-$	527.1554	527.1548	1.2	7.5
4	–1	–1	–1	$C_{35}H_{35}O_{13}^-$	663.2081	663.2083	–0.3	8.8
4	0	–2	0	$C_{36}H_{35}O_{14}^-$	691.2031	691.2032	–0.1	10.0

Table S-5. Assignment of MS^2 and MS^3 spectra shown in Figure S-11.

spectrum	structure assignment							mol.	*m/z*		rel. error	rel.
	CA	CO_2	$C_6H_6O_2$	CO	CH_2	H_2O	H_2	formula	exp.	theo.	[ppm]	Int.
MS^3 at *m/z* 161	2	–2	–1	0	0	–2	–1	$C_9H_6H^+$	115.0542	115.0542	0	8.2
	2	–2	–1	–1	0	0	–2	$C_9H_8OH^+$	133.0647	133.0648	–0.7	2.8
	2	–2	–1	0	0	–1	–1	$C_{10}H_6OH^+$	143.049	143.0491	–1.0	100.0
MS^3 at *m/z* 253	2	–2	0	–1	0	–2	–1	$C_{15}H_{12}O_2H^+$	207.0803	207.0804	–0.7	38.3
	2	–2	0	–1	0	–1	–1	$C_{15}H_{12}O_2H^+$	225.0909	225.091	–0.5	100.0
	2	–2	0	0	0	–2	–1	$C_{16}H_{10}O_3H^+$	235.0753	235.0754	–0.3	32.9
MS^2 at *m/z* 271	2	–2	–1	0	0	–2	–1	$C_9H_6H^+$	115.0542	115.0542	0	3.8
	1	–1	0	0	–1	0	–1	$C_{10}H_6O_2H^+$	123.044	123.0441	–0.8	2.8
	2	–2	–1	0	0	–1	–1	$C_{10}H_6OH^+$	143.049	143.0491	–1.0	20.3
	2	–2	–1	0	0	0	–1	$C_{10}H_8O_2H^+$	161.0596	161.0597	–0.7	100.0
	2	–2	0	0	0	–2	–1	$C_{16}H_{10}O_3H^+$	235.091	235.0754	66.5	3.1
	2	–2	0	0	0	–1	–1	$C_{16}H_{12}O_3H^+$	253.0858	253.0859	–0.5	22.7
MS^3 at *m/z* 297	1	–1	0	0	–1	0	–1	$C_7H_7O_2H^+$	123.044	123.0441	–0.5	20.8
	2	–2	–1	0	0	–1	–1	$C_{10}H_6OH^+$	143.049	143.0492	–1.4	20.3
	2	–2	–1	0	0	0	–1	$C_{10}H_8O_2H^+$	161.0596	161.0597	–0.7	100.0
	3	–3	–2	0	0	0	–1	$C_{12}H_{10}O_2H^+$	187.0753	187.0754	–0.3	49.5
	3	–3	–1	0	0	–1	–1	$C_{18}H_{14}O_3H^+$	279.1016	279.1021	–1.9	58.5
MS^3 at *m/z* 389	3	–3	0	–1	0	–1	–1	$C_{23}H_{20}O_4H^+$	361.1435	361.144	–1.3	100.0

MS² at *m/z* 407	1	–1	0	0	0	0	–1	$C_8H_8O_2H^+$	137.0596	137.0597	–0.8	4.3
	2	–2	–1	0	0	0	–1	$C_{10}H_8O_2H^+$	161.0596	161.0597	–0.7	6.1
	3	–3	–2	0	0	0	–1	$C_{12}H_{10}O_2H^+$	187.0753	187.0754	–0.3	1
	2	–2	0	0	–1	–1	0	$C_{15}H_{10}O_3H^+$	239.0702	239.0708	–2.6	0.1
	2	–2	0	0	–1	0	–1	$C_{15}H_{12}O_4H^+$	257.0807	257.0808	–0.4	15.2
	2	–2	0	0	0	0	–1	$C_{16}H_{14}O_4H^+$	271.0964	271.0965	–0.4	14.1
	3	–3	–1	0	0	0	–1	$C_{18}H_{16}O_4H^+$	297.1119	297.1121	–0.7	100.0
	3	–3	0	–1	0	–1	–1	$C_{23}H_{20}O_4H^+$	361.1435	361.144	–1.3	0.9
	3	–3	0	0	0	–2	–1	$C_{24}H_{18}O_4H^+$	371.1282	371.1283	–0.4	<0.1
	3	–3	0	0	0	–1	–1	$C_{24}H_{20}O_5H^+$	389.1384	389.1389	–1.3	10.7
MS² at *m/z* 543	2	–2	0	0	–1	0	–1	$C_{15}H_{12}O_4H^+$	257.0807	257.0808	–0.4	8.6
	2	–2	0	0	0	0	–1	$C_{16}H_{14}O_4H^+$	271.0964	271.0965	–0.4	29.1
	2	–2	0	0	0	0	0	$C_{16}H_{16}O_4H^+$	273.1121	273.1121	–0.1	7.7
	2	–2	–1	0	0	0	–1	$C_{18}H_{16}O_4H^+$	297.1121	297.1121	0	53.6
	3	–3	0	0	–1	0	–1	$C_{23}H_{20}O_6H^+$	393.1332	393.1333	–0.3	16.9
	3	–3	–1	0	0	0	–1	$C_{24}H_{22}O_6H^+$	407.1484	407.1489	–1.2	100.0
	3	–3	0	0	0	0	–2	$C_{26}H_{24}O_6H^+$	433.1644	433.1646	–0.5	95.1
	4	–4	0	0	0	–1	–1	$C_{32}H_{28}O_7H^+$	525.1907	525.1908	–0.3	14.6

Table S-6. Assignment of MS[2] spectra shown in Figure S-8B–C.

spectrum	structure assignment				mol.	*m/z*		rel. error	rel.
	CS	CO_2	$C_6H_6O_2$	H_2O	formula	exp.	theo.	[ppm]	Int.
MS² at *m/z* 285	1	–1	0	0	$C_8H_8OH^+$	121.0645	121.0648	–2.4	4.0
	2	–1	–1	0	$C_{11}H_8O_3H^+$	173.0593	173.0597	–2.4	2.5
	2	–1	–1	0	$C_{11}H_{10}O_3H^+$	191.0698	191.0703	–2.5	100.0
	2	–1	0	–1	$C_{17}H_{14}O_3H^+$	267.101	267.1016	–2.1	7.3
MS² at *m/z* 405	1	–1	0	0	$C_8H_8OH^+$	121.0645	121.0648	–2.4	48.1
	2	–1	–1	0	$C_{11}H_{10}O_3H^+$	191.0698	191.0703	–2.5	0.9
	2	–2	0	0	$C_{16}H_{16}O_2H^+$	241.1218	241.1223	–2.1	1.1
	3	–2	–1	–2	$C_{19}H_{14}O_2H^+$	275.1061	275.1067	–2.0	3.7
	3	–2	–1	–1	$C_{19}H_{16}O_3H^+$	293.1166	293.1172	–2.1	100.0
	3	–2	–1	0	$C_{19}H_{18}O_4H^+$	311.1273	311.1278	–1.6	12.3
	3	–2	0	–1	$C_{25}H_{22}O_4H^+$	387.1585	387.1591	–1.5	68.0

Table S-7. Assignment of MS[2] spectra shown in Figure S-10B–C.

spectrum	structure assignment			mol.	*m/z*		rel. error	rel.
	HC	CO_2	H_2O	formula	exp.	theo.	[ppm]	Int.
MS² at *m/z* 345	1	–1	0	$C_8H_9O_2^-$	137.0609	137.0608	0.7	8.2
	1	0	–1	$C_9H_7O_2^-$	163.0402	163.0401	0.8	30.3
	1	0	0	$C_9H_9O_4^-$	181.0507	181.0506	0.4	100.0
MS² at *m/z* 363	1	–1	0	$C_8H_9O_2^-$	137.0609	137.0608	0.7	5.3
	1	0	0	$C_9H_9O_4^-$	181.0507	181.0506	0.4	100.0

Table S-8. Assignment of signals obtained by HRMS analysis of (P1) measured using an APCI-orbitrap-MS instrument in positive ion mode.

	structure assignment					mol.	m/z		rel. error	rel.
CA	CO_2	$C_6H_6O_2$	H_2O	H_2	other	formula	exp.	theo.	[ppm]	intensity
1	–1	0	0	0	-	$C_8H_8O_2H^+$	137.0595	137.0597	-1.5	10.9
2	–2	–1	–1	0	-	$C_{10}H_8OH^+$	145.0646	145.0648	-1.3	10.2
2	–2	–1	0	0	–CH_2	$C_9H_8O_2H^+$	149.0595	149.0597	-1.4	4.7
2	–2	–1	0	0	-	$C_{10}H_{10}O_2H^+$	163.0750	163.0754	-2.5	100.0
2	–2	0	0	–2	-	$C_{16}H_{12}O_4H^+$	269.0806	269.0808	-0.7	2.6
2	–2	0	0	–1	-	$C_{16}H_{14}O_4H^+$	271.0961	271.0965	-1.5	17.9

Table S-9. Assignment of signals obtained by HRMS analysis of (P1) at 220 °C for 5 min measured using an APCI-orbitrap-MS instrument in positive ion mode.

	structure assignment					mol.	m/z		rel. error	rel.
CA	CO_2	$C_6H_6O_2$	H_2O	H_2	other	formula	exp.	theo.	[ppm]	intensity
2	–2	–1	0	–1	-	$C_{10}H_8O_2H^+$	161.0600	161.0597	1.8	1.8
2	–2	–1	0	0	-	$C_{10}H_{10}O_2H^+$	163.0755	163.0754	0.6	33.5
2	–2	0	0	–2	–CH_2	$C_{15}H_{10}O_4H^+$	255.0655	255.0652	1.2	1.0
2	–2	0	0	–1	–CH_2	$C_{15}H_{12}O_4H^+$	257.0812	257.0808	1.6	3.6
2	–2	0	0	0	–CH_2	$C_{15}H_{14}O_4H^+$	259.0969	259.0965	1.6	2.3
2	–2	0	0	–3	-	$C_{16}H_{10}O_4H^+$	267.0657	267.0652	1.9	4.8
2	–2	0	0	–2	-	$C_{16}H_{12}O_4H^+$	269.0813	269.0808	1.7	28.7
2	–2	0	0	–1	-	$C_{16}H_{14}O_4H^+$	271.0967	271.0965	0.8	100.0
2	–2	0	1	–4	-	$C_{16}H_{10}O_5H^+$	283.0605	283.0601	1.4	2.7
2	–2	0	1	–3	-	$C_{16}H_{12}O_5H^+$	285.0761	285.0758	1.2	15.9
2	–2	0	1	–2	-	$C_{16}H_{14}O_5H^+$	287.0918	287.0914	1.4	9.4
4	–4	–2	0	0	–2×CH_2	$C_{18}H_{16}O_4H^+$	297.1126	297.1121	1.7	2.8
4	–4	–2	0	1	–2×CH_2	$C_{18}H_{18}O_4H^+$	299.1282	299.1278	1.4	1.2

4	–4	–2	1	–2	–2×CH_2	$C_{18}H_{14}O_5H^+$	311.0919	311.0914	1.6	1.4
4	–4	–2	1	–1	–2×CH_2	$C_{18}H_{16}O_5H^+$	313.1075	313.1071	1.4	3.2
3	–3	0	0	–4	-	$C_{24}H_{16}O_6H^+$	401.1024	401.1020	1.1	1.5
3	–3	0	0	–3	-	$C_{24}H_{18}O_6H^+$	403.1181	403.1176	1.2	5.2
3	–3	0	0	–2	-	$C_{24}H_{20}O_6H^+$	405.1337	405.1333	1.1	12.9
3	–3	0	0	–1	-	$C_{24}H_{22}O_6H^+$	407.1493	407.1489	1.0	3.8
3	–3	0	1	–4	-	$C_{24}H_{18}O_7H^+$	419.1129	419.1125	1.0	1.7
3	–3	0	1	–3	-	$C_{24}H_{20}O_7H^+$	421.1285	421.1282	0.8	1.1
4	–4	–1	0	–4	-	$C_{26}H_{20}O_6H^+$	429.1336	429.1333	0.8	1.2
4	–4	–1	0	–3	-	$C_{26}H_{22}O_6H^+$	431.1493	431.1489	0.9	3.9
4	–4	–1	0	–2	-	$C_{26}H_{24}O_6H^+$	433.1649	433.1646	0.8	7.3
4	–4	–1	1	–4	-	$C_{26}H_{22}O_7H^+$	447.1441	447.1438	0.6	1.0
4	–4	0	0	–5	-	$C_{32}H_{22}O_8H^+$	535.1390	535.1387	0.5	<0.1
4	–4	0	0	–4	-	$C_{32}H_{24}O_8H^+$	537.1548	537.1544	0.8	0.1
4	–4	0	0	–3	-	$C_{32}H_{26}O_8H^+$	539.1703	539.1700	0.5	0.5
4	–4	0	0	–2	-	$C_{32}H_{28}O_8H^+$	541.1861	541.1857	0.8	0.5
4	–4	0	0	–1	-	$C_{32}H_{30}O_8H^+$	543.2018	543.2013	0.8	0.1

Table S-10. Assignment of signals obtained by HRMS analysis of (P1 + CA) at 220 °C for 5 min measured using an APCI-orbitrap-MS instrument in positive ion mode.

	structure assignment					mol.	*m/z*		rel. error	rel.
CA	CO_2	$C_6H_6O_2$	H_2O	H_2	other	formula	exp.	theo.	[ppm]	intensity
1	–1	0	1	–1	-	$C_8H_8O_3H^+$	153.0550	153.0546	2.5	2.0
2	–2	–1	0	–1	-	$C_{10}H_8O_2H^+$	161.0600	161.0597	1.8	3.9
2	–2	–1	0	0	-	$C_{10}H_{10}O_2H^+$	163.0756	163.0754	1.2	24.3
2	–2	–1	1	–2	-	$C_{10}H_8O_3H^+$	177.0549	177.0546	1.6	3.8
2	–2	–1	1	–1	-	$C_{10}H_{10}O_3H^+$	179.0705	179.0703	1.3	8.5
2	–2	0	–1	0	–CH_2	$C_{10}H_{14}O_3H^+$	243.1019	243.1016	1.4	1.1

2	–2	0	0	–3	–CH_2	$C_{15}H_{10}O_4H^+$	255.0655	255.0652	1.2	1.1
2	–2	0	–1	–1	-	$C_{16}H_{14}O_3H^+$	255.1019	255.1016	1.3	1.8
2	–2	0	0	–2	–CH_2	$C_{15}H_{12}O_4H^+$	257.0812	257.0808	1.4	5.0
2	–2	0	0	–1	–CH_2	$C_{15}H_{14}O_4H^+$	259.0969	259.0965	1.6	2.3
2	–2	0	0	–4	-	$C_{16}H_{10}O_4H^+$	267.0656	267.0652	1.5	2.0
2	–2	0	0	–2	-	$C_{16}H_{12}O_4H^+$	269.0813	269.0808	1.9	23.1
2	–2	0	0	–1	-	$C_{16}H_{14}O_4H^+$	271.0967	271.0965	0.7	98.6
2	–2	0	1	–5	-	$C_{16}H_{10}O_5H^+$	283.0605	283.0610	-1.8	2.4
2	–2	0	1	–4	-	$C_{16}H_{12}O_5H^+$	285.0761	285.0758	1.2	26.4
2	–2	0	1	–3	-	$C_{16}H_{14}O_5H^+$	287.0918	287.0914	1.4	13.0
3	–3	–1	0	–4	-	$C_{18}H_{12}O_4H^+$	293.0813	293.0808	1.6	1.8
3	–3	–1	0	–3	-	$C_{18}H_{14}O_4H^+$	295.0969	295.0965	1.4	1.6
3	–3	–1	0	–2	-	$C_{18}H_{16}O_4H^+$	297.1126	297.1121	1.5	6.8
3	–3	–1	0	–1	-	$C_{18}H_{18}O_4H^+$	299.1282	299.1278	1.4	12.3
2	–2	0	2	–4	-	$C_{16}H_{14}O_5H^+$	303.0868	303.0863	1.6	2.9
3	–3	–1	1	–4	-	$C_{18}H_{14}O_5H^+$	311.0919	311.0914	1.6	1.2
3	–3	–1	1	–3	-	$C_{18}H_{16}O_5H^+$	313.1076	313.1071	1.8	3.0
3	–3	0	0	–5	-	$C_{24}H_{14}O_6H^+$	399.0868	399.0863	1.2	1.4
3	–3	0	0	–4	-	$C_{24}H_{16}O_6H^+$	401.1025	401.1020	1.3	3.7
3	–3	0	0	–3	-	$C_{24}H_{18}O_6H^+$	403.1181	403.1176	1.2	11.5
3	–3	0	0	–2	-	$C_{24}H_{20}O_6H^+$	405.1337	405.1333	1.1	30.0
3	–3	0	0	–1	-	$C_{24}H_{22}O_6H^+$	407.1493	407.1489	1.0	14.2
3	–3	0	1	–4	-	$C_{24}H_{18}O_7H^+$	419.1129	419.1125	0.9	2.6
3	–3	0	1	–3	-	$C_{24}H_{20}O_7H^+$	421.1286	421.1282	1.0	2.6
4	–4	–1	0	–4	-	$C_{26}H_{20}O_6H^+$	429.1336	429.1333	0.8	1.5
4	–4	–1	0	–3	-	$C_{26}H_{22}O_6H^+$	431.1493	431.1489	0.9	3.7
4	–4	–1	0	–2	-	$C_{26}H_{24}O_6H^+$	433.1649	433.1646	0.8	6.4
4	–4	–1	0	–1	-	$C_{26}H_{26}O_6H^+$	435.1805	435.1802	0.6	2.6

4	–4	–1	1	–4	-	$C_{26}H_{22}O_7H^+$	447.1442	447.1438	0.8	1.1
4	–4	0	0	–4	-	$C_{32}H_{24}O_8H^+$	537.1550	537.1544	1.1	0.5
4	–4	0	0	–3	-	$C_{32}H_{26}O_8H^+$	539.1704	539.1700	0.7	1.3
4	–4	0	0	–2	-	$C_{32}H_{28}O_8H^+$	541.1861	541.1857	0.8	2.9
4	–4	0	0	–1	-	$C_{32}H_{30}O_8H^+$	543.2018	543.2013	0.8	2.3

Table S-11. Assignment of signals obtained by HRMS analysis of (P3) measured using an APCI-orbitrap-MS instrument in positive ion mode.

	structure assignment					mol.	*m/z*		rel. error	rel.
**CA**	**CO_2**	**$C_6H_6O_2$**	**H_2O**	**H_2**	**other**	**formula**	**exp.**	**theo.**	**[ppm]**	**intensity**
1	–1	0	0	1	–2× CH_2	$C_6H_6O_2H^+$	111.0440	111.0441	-0.7	0.7
2	–2	0	–2	1	–$C_7H_6O_2$	$C_9H_8H^+$	117.0698	117.0699	-1.1	2.4
1	–1	0	–1	0	-	$C_8H_6OH^+$	119.0490	119.0491	-0.9	1.3
1	–1	0	0	0	–CH_2	$C_7H_6O_2H^+$	123.0439	123.0441	-1.2	1.6
1	–1	0	0	0	-	$C_8H_8O_2H^+$	137.0595	137.0597	-1.3	41.9
1	–1	0	1	0	–CH_2	$C_7H_8O_3H^+$	139.0388	139.0390	-1.3	9.3
2	–2	–1	–1	0	-	$C_{10}H_8OH^+$	145.0646	145.0648	-1.0	19.1
2	–2	–1	0	0	–CH_2	$C_9H_8O_2H^+$	149.0596	149.0597	-0.9	12.4
1	–1	0	1	–1	-	$C_8H_8O_3H^+$	153.0545	153.0546	-0.7	1.1
2	–2	–1	0	0	-	$C_{10}H_{10}O_2H^+$	163.0751	163.0754	-1.8	100.0
2	–2	0	0	0	–C_3H_6	$C_{13}H_{10}O_4H^+$	231.0650	231.0652	-0.8	1.1
2	–2	0	0	–1	–CH_2	$C_{15}H_{12}O_4H^+$	257.0807	257.0808	-0.5	2.2
2	–2	0	0	0	–CH_2	$C_{15}H_{14}O_4H^+$	259.0963	259.0965	-0.6	1.4
2	–2	0	0	–2	-	$C_{16}H_{12}O_4H^+$	269.0807	269.0808	-0.5	2.4
2	–2	0	0	–1	-	$C_{16}H_{14}O_4H^+$	271.0963	271.0965	-0.8	23.6
2	–2	0	0	0	-	$C_{16}H_{16}O_4H^+$	273.1119	273.1121	-0.7	6.3
2	–2	0	1	–3	-	$C_{16}H_{12}O_5H^+$	285.0756	285.0758	-0.4	1.0
3	–3	0	0	–1	–$C_7H_6O_2$	$C_{17}H_{16}O_4H^+$	285.1120	285.1121	-0.5	0.7

2	–2	0	1	–2	-	$C_{16}H_{14}O_5H^+$	287.0912	287.0914	-0.6	0.6
3	–3	–1	0	–4	-	$C_{18}H_{12}O_4H^+$	293.0807	293.0808	-0.6	0.5
3	–3	–1	0	–3	-	$C_{18}H_{16}O_4H^+$	297.1120	297.1121	-0.5	13.9
3	–3	–1	0	–2	-	$C_{18}H_{18}O_4H^+$	299.1276	299.1278	-0.6	8.4
4	–4	–2	0	0	-	$C_{20}H_{20}O_4H^+$	325.1433	325.1434	-0.4	5.3
3	–3	0	0	–3	-	$C_{24}H_{18}O_6H^+$	403.1173	403.1176	-0.8	3.7
3	–3	0	0	–2	-	$C_{24}H_{20}O_6H^+$	405.1330	405.1333	-0.7	4.0
3	–3	0	0	–1	-	$C_{24}H_{22}O_6H^+$	407.1486	407.1489	-0.8	6.9
3	–3	0	0	0	-	$C_{24}H_{24}O_6H^+$	409.1643	409.1646	-0.7	0.9
4	–4	–1	0	–2	-	$C_{26}H_{24}O_6H^+$	433.1642	433.1646	-0.9	2.0
4	–4	0	1	–3	$–C_7H_6O_2$	$C_{25}H_{22}O_7H^+$	435.1434	435.1438	-0.9	0.8
4	–4	–1	0	–1	-	$C_{26}H_{26}O_6H^+$	435.1798	435.1802	-0.9	7.8
4	–4	0	0	–2	-	$C_{32}H_{28}O_8H^+$	541.1852	541.1857	-0.9	1.1
4	–4	0	0	–1	-	$C_{32}H_{30}O_8H^+$	543.2009	543.2013	-0.8	1.3

Table S-12. Assignment of signals obtained by HRMS analysis of (P3) at 220 °C for 5 min measured using an APCI-orbitrap-MS instrument in positive ion mode.

	structure assignment					mol.	*m/z*		rel. error	rel.
**CA**	**CO_2**	**$C_6H_6O_2$**	**H_2O**	**H_2**	**other**	**formula**	**exp.**	**theo.**	**[ppm]**	**intensity**
1	–1	0	1	–1	-	$C_8H_8O_3H^+$	153.0546	153.0546	0.2	1.9
2	–2	–1	0	–1	-	$C_{10}H_8O_2H^+$	161.0597	161.0597	-0.2	1.2
2	–2	–1	0	0	-	$C_{10}H_{10}O_2H^+$	163.0753	163.0754	-0.9	41.0
2	–2	–1	1	–2	-	$C_{10}H_8O_3H^+$	177.0546	177.0546	-0.3	4.2
2	–2	–1	1	–1	-	$C_{10}H_{10}O_3H^+$	179.0702	179.0703	-0.3	3.5
2	–2	0	0	0	$–C_3H_6$	$C_{13}H_{10}O_4H^+$	231.0651	231.0652	-0.3	3.7
2	–2	0	0	–1	$–2\times CH_2$	$C_{14}H_{10}O_4H^+$	243.0651	243.0652	-0.4	1.4
2	–2	0	0	–1	$–CH_2$	$C_{15}H_{12}O_4H^+$	257.0808	257.0808	-0.2	3.3
2	–2	0	0	–1	$–CH_2$	$C_{15}H_{14}O_4H^+$	259.0964	259.0965	-0.5	1.1

2	–2	0	0	–2	-	$C_{16}H_{12}O_4H^+$	269.0808	269.0808	-0.1	13.2
3	–3	0	–1	0	$–C_7H_6O_2$	$C_{17}H_{16}O_3H^+$	269.1172	269.1172	-0.1	0.7
2	–2	0	0	–1	-	$C_{16}H_{14}O_4H^+$	271.0963	271.0965	-0.6	100.0
2	–2	0	0	–1	-	$C_{16}H_{16}O_4H^+$	273.1120	273.1121	-0.4	3.2
2	–2	0	1	–4	-	$C_{16}H_{10}O_5H^+$	283.0600	283.0601	-0.2	1.5
3	–3	0	0	–2	$–C_7H_6O_2$	$C_{17}H_{14}O_4H^+$	283.0965	283.0965	0.0	2.7
2	–2	0	0	–2	-	$C_{16}H_{12}O_5H^+$	285.0757	285.0758	-0.3	36.6
3	–3	–1	0	0	$–CH_2$	$C_{17}H_{16}O_4H^+$	285.1121	285.1120	0.2	2.2
2	–2	0	1	–2	-	$C_{16}H_{14}O_5H^+$	287.0914	287.0914	-0.1	14.1
3	–3	–1	0	–5	-	$C_{18}H_{12}O_4H^+$	293.0808	293.0808	0.0	5.3
3	–3	–1	0	–4	-	$C_{18}H_{14}O_4H^+$	295.0965	295.0965	-0.1	5.7
3	–3	–1	0	–3	-	$C_{18}H_{16}O_4H^+$	297.1121	297.1121	0.0	16.1
3	–3	–1	0	–2	-	$C_{18}H_{18}O_4H^+$	299.1277	299.1278	-0.2	10.6
2	–2	0	2	–4	-	$C_{16}H_{12}O_6H^+$	301.0706	301.0707	-0.2	1.0
2	–2	0	2	–3	-	$C_{16}H_{14}O_6H^+$	303.0863	303.0863	0.0	9.8
3	–3	–1	1	–1	-	$C_{18}H_{14}O_5H^+$	311.0914	311.0914	0.0	1.1
4	–4	–1	0	–1	$–C_7H_6O_2$	$C_{19}H_{18}O_4H^+$	311.1278	311.1278	0.2	0.8
3	–3	–1	1	–2	-	$C_{18}H_{16}O_5H^+$	313.1071	313.1071	0.0	3.6
4	–4	–2	0	0	-	$C_{20}H_{20}O_4H^+$	325.1434	325.1434	-0.1	4.2
3	–3	0	0	0	$–3\times CH_2$	$C_{21}H_{18}O_6H^+$	367.1176	367.1176	-0.1	1.2
3	–3	0	0	–1	$–2\times CH_2$	$C_{22}H_{18}O_6H^+$	379.1176	379.1176	0.0	1.4
3	–3	0	0	–3	$–CH_2$	$C_{23}H_{16}O_6H^+$	389.1019	389.1020	-0.3	2.6
3	–3	0	0	–2	$–CH_2$	$C_{23}H_{18}O_6H^+$	391.1175	391.1176	-0.3	3.5
3	–3	0	0	–1	$–CH_2$	$C_{23}H_{20}O_6H^+$	393.1331	393.1333	-0.4	2.0
3	–3	0	0	–5	-	$C_{24}H_{14}O_6H^+$	399.0861	399.0856	1.3	6.1
3	–3	0	0	–4	-	$C_{24}H_{16}O_6H^+$	401.1018	401.1020	-0.5	6.6
3	–3	0	0	–3	-	$C_{24}H_{18}O_6H^+$	403.1174	403.1176	-0.5	28.5
3	–3	0	0	–2	-	$C_{24}H_{20}O_6H^+$	405.1330	405.1333	-0.6	42.0

3	–3	0	0	–1	-	$C_{24}H_{22}O_6H^+$	407.1487	407.1489	-0.6	16.8
3	–3	0	0	0	-	$C_{24}H_{24}O_6H^+$	409.1644	409.1646	-0.5	2.2
3	–3	0	1	–5	-	$C_{24}H_{16}O_7H^+$	417.0967	417.0969	-0.4	1.3
4	–4	0	0	–3	$–C_7H_6O_2$	$C_{25}H_{20}O_6H^+$	417.1330	417.1333	-0.5	0.9
3	–3	0	1	–4	-	$C_{24}H_{18}O_7H^+$	419.1123	419.1125	-0.4	2.5
3	–3	0	1	–3	-	$C_{24}H_{20}O_7H^+$	421.1280	421.1282	-0.6	2.6
4	–4	–1	0	–4	-	$C_{26}H_{18}O_6H^+$	427.1173	427.1176	-0.7	2.1
4	–4	–1	0	–3	-	$C_{26}H_{20}O_6H^+$	429.1330	429.1333	-0.6	5.8
4	–4	–1	0	–2	-	$C_{26}H_{22}O_6H^+$	431.1486	431.1489	-0.6	13.4
4	–4	–1	0	–1	-	$C_{26}H_{24}O_6H^+$	433.1643	433.1646	-0.7	23.3
4	–4	0	1	–3	$–C_7H_6O_2$	$C_{25}H_{22}O_7H^+$	435.1436	435.1438	-0.5	1.0
4	–4	–1	0	0	-	$C_{26}H_{26}O_6H^+$	435.1799	435.1802	-0.7	10.1
4	–4	–1	1	–4	-	$C_{26}H_{20}O_7H^+$	445.1279	445.1282	-0.7	1.5
4	–4	–1	1	–3	-	$C_{26}H_{22}O_7H^+$	447.1436	447.1438	-0.5	2.1
4	–4	–1	1	–2	-	$C_{26}H_{24}O_7H^+$	449.1592	449.1595	-0.6	1.2
5	–5	–2	0	–1	-	$C_{28}H_{28}O_6H^+$	459.1799	459.1802	-0.6	0.8
5	–5	–2	0	0	-	$C_{28}H_{26}O_6H^+$	461.1955	461.1959	-0.7	1.2
4	–4	0	0	–5	-	$C_{32}H_{22}O_8H^+$	535.1385	535.1387	-0.4	2.1
4	–4	0	0	–4	-	$C_{32}H_{24}O_8H^+$	537.1541	537.1538	0.6	5.3
4	–4	0	0	–3	-	$C_{32}H_{26}O_8H^+$	539.1697	539.1694	0.5	12.3
4	–4	0	0	–2	-	$C_{32}H_{28}O_8H^+$	541.1853	541.1857	-0.6	20.5
4	–4	0	0	–2	-	$C_{32}H_{30}O_8H^+$	543.2010	543.2013	-0.7	17.9
4	–4	0	0	–1	-	$C_{32}H_{32}O_8H^+$	545.2166	545.2170	-0.7	0.8
4	–4	0	1	–5	-	$C_{32}H_{24}O_9H^+$	553.1490	553.1493	-0.6	0.8
4	–4	0	1	–4	-	$C_{32}H_{24}O_9H^+$	555.1646	555.1650	-0.7	1.1
4	–4	0	1	–3	-	$C_{32}H_{24}O_9H^+$	557.1801	557.1806	-0.9	0.8
5	–5	–1	0	–4	-	$C_{34}H_{26}O_8H^+$	563.1697	563.1700	-0.5	0.9
5	–5	–1	0	–3	-	$C_{34}H_{28}O_8H^+$	565.1854	565.1857	-0.6	1.9

5	–5	–1	0	–2	-	$C_{34}H_{30}O_8H^+$	567.2010	567.2013	-0.6	4.0
5	–5	–1	0	–1	-	$C_{34}H_{32}O_8H^+$	569.2166	569.2163	0.5	4.7
5	–5	–1	0	0	-	$C_{34}H_{32}O_8H^+$	571.2323	571.2326	-0.6	2.8
5	–5	0	0	–3	-	$C_{40}H_{34}O_{10}H^+$	675.2222	675.2225	-0.5	1.0
5	–5	0	0	–2	-	$C_{40}H_{36}O_{10}H^+$	677.2379	677.2381	-0.3	1.0
5	–5	0	0	–1	-	$C_{40}H_{38}O_{10}H^+$	679.2537	679.2538	-0.1	0.8

Table S-13. Assignment of signals obtained by HRMS analysis of (P3 + CA) at 220 °C for 5 min measured using an APCI-orbitrap-MS instrument in positive ion mode.

	structure assignment					mol.	*m/z*		rel. error	rel.
**CA**	**CO_2**	**$C_6H_6O_2$**	**H_2O**	**H_2**	**other**	**formula**	**exp.**	**theo.**	**[ppm]**	**intensity**
1	–1	0	1	–1	-	$C_8H_8O_3H^+$	153.0546	153.0546	-0.3	5.4
2	–2	–1	0	–1	-	$C_{10}H_8O_2H^+$	161.0596	161.0597	-0.7	0.9
2	–2	–1	0	0	-	$C_{10}H_{10}O_2H^+$	163.0752	163.0754	-1.2	48.7
2	–2	–1	1	–2	-	$C_{10}H_8O_3H^+$	177.0545	177.0546	-0.6	2.3
2	–2	–1	1	–1	-	$C_{10}H_{10}O_3H^+$	179.0701	179.0703	-0.8	8.2
2	–2	0	0	0	$-C_3H_6$	$C_{13}H_{10}O_4H^+$	231.0651	231.0652	-0.6	3.2
2	–2	0	0	–1	$-2\times CH_2$	$C_{14}H_{10}O_4H^+$	243.0650	243.0652	-0.7	2.4
2	–2	0	0	–2	$-CH_2$	$C_{15}H_{10}O_4H^+$	255.0650	255.0652	-0.7	0.9
2	–2	0	0	–1	$-CH_2$	$C_{15}H_{12}O_4H^+$	257.0807	257.0808	-0.6	3.7
2	–2	0	0	–1	$-CH_2$	$C_{15}H_{14}O_4H^+$	259.0963	259.0965	-0.8	1.0
2	–2	0	0	–3	-	$C_{16}H_{10}O_4H^+$	267.0650	267.0652	-0.7	1.7
2	–2	0	0	–2	-	$C_{16}H_{12}O_4H^+$	269.0807	269.0808	-0.6	16.3
2	–2	0	0	–1	-	$C_{16}H_{14}O_4H^+$	271.0963	271.0965	-0.8	74.4
2	–2	0	0	–1	-	$C_{16}H_{16}O_4H^+$	273.1119	273.1121	-0.6	8.4
2	–2	0	1	–4	-	$C_{16}H_{10}O_5H^+$	283.0600	283.0601	-0.4	3.0
3	–3	0	0	–2	$-C_7H_6O_2$	$C_{17}H_{14}O_4H^+$	283.0964	283.0965	-0.3	1.9
2	–2	0	0	–2	-	$C_{16}H_{12}O_5H^+$	285.0756	285.0758	-0.6	40.0

3	–3	–1	0	0	$-CH_2$	$C_{17}H_{16}O_4H^+$	285.1120	285.1120	0.0	2.6
2	–2	0	1	–2	-	$C_{16}H_{14}O_5H^+$	287.0913	287.0914	-0.5	8.8
2	–2	0	1	–1	-	$C_{16}H_{16}O_5H^+$	289.1069	289.1071	-0.5	1.3
3	–3	–1	0	–5	-	$C_{18}H_{12}O_4H^+$	293.0807	293.0808	-0.4	8.5
3	–3	–1	0	–4	-	$C_{18}H_{14}O_4H^+$	295.0964	295.0965	-0.4	7.3
3	–3	–1	0	–3	-	$C_{18}H_{16}O_4H^+$	297.1120	297.1121	-0.4	18.9
3	–3	–1	0	–2	-	$C_{18}H_{18}O_4H^+$	299.1276	299.1278	-0.5	21.0
2	–2	0	2	–4	-	$C_{16}H_{12}O_6H^+$	301.0705	301.0707	-0.6	0.8
2	–2	0	2	–3	-	$C_{16}H_{14}O_6H^+$	303.0862	303.0863	-0.4	8.1
3	–3	–1	1	–1	-	$C_{18}H_{14}O_5H^+$	311.0913	311.0914	-0.3	1.3
4	–4	–1	0	–1	$-C_7H_6O_2$	$C_{19}H_{18}O_4H^+$	311.1277	311.1278	-0.3	0.9
3	–3	–1	1	–2	-	$C_{18}H_{16}O_5H^+$	313.1070	313.1071	-0.3	3.4
3	–3	–1	1	–1	-	$C_{18}H_{18}O_5H^+$	315.1226	315.1227	-0.4	1.0
4	–4	–2	0	0	-	$C_{20}H_{20}O_4H^+$	325.1432	325.1434	-0.6	3.5
3	–3	0	0	0	$-3\times CH_2$	$C_{21}H_{18}O_6H^+$	367.1175	367.1176	-0.4	0.9
3	–3	0	0	–3	$-2\times CH_2$	$C_{22}H_{14}O_6H^+$	375.0862	375.0863	-0.3	1.4
3	–3	0	0	–1	$-2\times CH_2$	$C_{22}H_{18}O_6H^+$	379.1174	379.1176	-0.5	2.4
3	–3	0	0	–4	$-CH_2$	$C_{23}H_{16}O_6H^+$	387.0860	387.0863	-0.8	0.8
3	–3	0	0	–3	$-CH_2$	$C_{23}H_{16}O_6H^+$	389.1017	389.1020	-0.7	3.4
3	–3	0	0	–2	$-CH_2$	$C_{23}H_{18}O_6H^+$	391.1173	391.1176	-0.8	5.1
3	–3	0	0	–1	$-CH_2$	$C_{23}H_{20}O_6H^+$	393.1329	393.1333	-1.0	2.0
3	–3	0	0	–5	-	$C_{24}H_{14}O_6H^+$	399.0860	399.0856	0.9	7.2
3	–3	0	0	–4	-	$C_{24}H_{16}O_6H^+$	401.1016	401.1020	-0.8	14.4
3	–3	0	0	–3	-	$C_{24}H_{18}O_6H^+$	403.1172	403.1176	-0.9	69.7
3	–3	0	0	–2	-	$C_{24}H_{20}O_6H^+$	405.1328	405.1333	-1.0	100.0
3	–3	0	0	–1	-	$C_{24}H_{22}O_6H^+$	407.1485	407.1489	-1.0	34.3
3	–3	0	0	0	-	$C_{24}H_{24}O_6H^+$	409.1642	409.1646	-0.9	4.4
3	–3	0	1	–5	-	$C_{24}H_{16}O_7H^+$	417.0965	417.0969	-1.0	3.1

3	–3	0	1	–4	-	$C_{24}H_{18}O_7H^+$	419.1121	419.1125	-0.9	5.1
4	–4	0	0	–2	$–C_7H_6O_2$	$C_{25}H_{22}O_6H^+$	419.1485	419.1489	-1.0	1.0
3	–3	0	1	–3	-	$C_{24}H_{20}O_7H^+$	421.1278	421.1282	-1.0	5.7
3	–3	0	1	–2	-	$C_{24}H_{22}O_7H^+$	423.1434	423.1438	-1.0	1.3
4	–4	–1	0	–4	-	$C_{26}H_{18}O_6H^+$	427.1172	427.1176	-1.0	2.0
4	–4	–1	0	–3	-	$C_{26}H_{20}O_6H^+$	429.1328	429.1333	-1.0	6.8
4	–4	–1	0	–2	-	$C_{26}H_{22}O_6H^+$	431.1484	431.1489	-1.1	12.6
4	–4	–1	0	–1	-	$C_{26}H_{24}O_6H^+$	433.1641	433.1646	-1.1	18.6
4	–4	0	1	–3	$–C_7H_6O_2$	$C_{25}H_{22}O_7H^+$	435.1434	435.1438	-1.0	1.6
4	–4	–1	0	0	-	$C_{26}H_{26}O_6H^+$	435.1797	435.1802	-1.0	12.7
4	–4	–1	1	–4	-	$C_{26}H_{20}O_7H^+$	445.1277	445.1282	-1.2	1.5
4	–4	–1	1	–3	-	$C_{26}H_{22}O_7H^+$	447.1433	447.1438	-1.1	2.0
4	–4	–1	1	–2	-	$C_{26}H_{24}O_7H^+$	449.1589	449.1595	-1.3	1.0
5	–5	–2	0	0	-	$C_{28}H_{26}O_6H^+$	461.1954	461.1959	-1.0	1.0
4	–4	0	0	–5	-	$C_{32}H_{22}O_8H^+$	535.1383	535.1387	-0.9	2.2
4	–4	0	0	–4	-	$C_{32}H_{24}O_8H^+$	537.1539	537.1538	0.2	4.8
4	–4	0	0	–3	-	$C_{32}H_{26}O_8H^+$	539.1695	539.1694	0.2	13.0
4	–4	0	0	–2	-	$C_{32}H_{28}O_8H^+$	541.1851	541.1857	-1.1	21.5
4	–4	0	0	–2	-	$C_{32}H_{30}O_8H^+$	543.2008	543.2013	-1.0	14.5
4	–4	0	0	–1	-	$C_{32}H_{32}O_8H^+$	545.2163	545.2170	-1.4	1.6
4	–4	0	1	–4	-	$C_{32}H_{24}O_9H^+$	555.1643	555.1650	-1.1	0.8
5	–5	–1	0	–3	-	$C_{34}H_{28}O_8H^+$	565.1852	565.1857	-0.8	1.4
5	–5	–1	0	–2	-	$C_{34}H_{30}O_8H^+$	567.2008	567.2013	-1.0	2.8
5	–5	–1	0	–1	-	$C_{34}H_{32}O_8H^+$	569.2164	569.2163	0.2	3.5
5	–5	–1	0	0	-	$C_{34}H_{32}O_8H^+$	571.2321	571.2326	-0.9	1.7
5	–5	0	0	–2	-	$C_{40}H_{36}O_{10}H^+$	677.2374	677.2381	-1.1	0.7
5	–5	0	0	–1	-	$C_{40}H_{38}O_{10}H^+$	679.2535	679.2538	-0.4	0.7

Table S-14. Assignment of signals obtained by HRMS analysis of (P2) measured using an APCI-orbitrap-MS instrument in positive ion mode.

	structure assignment					mol.	*m/z*		rel. error	rel.
CA	CO_2	$C_6H_6O_2$	H_2O	H_2	other	formula	exp.	theo.	[ppm]	intensity
2	–2	0	–2	1	$-C_7H_6O_2$	$C_9H_8H^+$	117.0697	117.0699	-1.7	1.5
1	–1	0	0	0	-	$C_8H_9O_2H^+$	137.0595	137.0597	-1.5	22.6
2	–2	–1	–1	0	-	$C_{10}H_8OH^+$	145.0646	145.0648	-1.3	10.7
2	–2	–1	0	0	$-CH_2$	$C_9H_8O_2H^+$	149.0596	149.0597	-0.7	5.3
2	–2	–1	0	0	-	$C_{10}H_{10}O_2H^+$	163.0751	163.0754	-1.8	100.0
2	–2	0	0	0	$-C_5H_6$	$C_{13}H_{10}O_4H^+$	231.0650	231.0652	-0.9	1.9
2	–2	0	0	–2	-	$C_{16}H_{12}O_4H^+$	269.0807	269.0808	-0.5	3.1
2	–2	0	0	–1	-	$C_{16}H_{14}O_4H^+$	271.0967	271.0965	0.8	26.9
2	–2	0	1	–3	-	$C_{16}H_{12}O_5H^+$	285.0756	285.0758	-0.5	2.5
2	–2	0	1	–2	-	$C_{16}H_{14}O_5H^+$	287.0912	287.0914	-0.7	0.9
3	–3	–1	0	–3	-	$C_{18}H_{16}O_4H^+$	297.1119	297.1121	-0.7	0.9
3	–3	–1	0	–2	-	$C_{18}H_{18}O_4H^+$	299.1276	299.1278	-0.6	2.0
3	–3	0	0	–2	-	$C_{24}H_{20}O_6H^+$	405.1329	405.1333	-0.9	1.6
3	–3	0	0	–1	-	$C_{24}H_{22}O_6H^+$	407.1485	407.1489	-1.0	2.1

Table S-15. Assignment of signals obtained by HRMS analysis of (P2) at 220 °C for 5 min measured using an APCI-orbitrap-MS instrument in positive ion mode.

	structure assignment					mol.	*m/z*		rel. error	rel.
CA	CO_2	$C_6H_6O_2$	H_2O	H_2	other	formula	exp.	theo.	[ppm]	intensity
2	–2	–1	0	0	-	$C_{10}H_{10}O_2H^+$	163.0753	163.0754	-0.6	100.0
2	–2	–1	1	–2	-	$C_{10}H_8O_3H^+$	177.0545	177.0546	-0.7	5.0
2	–2	–1	1	–1	-	$C_{10}H_{10}O_3H^+$	179.0702	179.0703	-0.4	5.1
2	–2	0	–1	–1	-	$C_{16}H_{14}O_3H^+$	255.1014	255.1016	-0.7	0.9
2	–2	0	0	–1	$–CH_2$	$C_{15}H_{12}O_4H^+$	257.0807	257.0808	-0.5	2.4
2	–2	0	0	–3	-	$C_{16}H_{10}O_4H^+$	267.0651	267.0652	-0.3	2.3
2	–2	0	0	–2	-	$C_{16}H_{12}O_4H^+$	269.0808	269.0808	-0.1	18.5
2	–2	0	0	–1	-	$C_{16}H_{14}O_4H^+$	271.0962	271.0965	-1.1	100.0
2	–2	0	1	–4	-	$C_{16}H_{10}O_5H^+$	283.0600	283.0601	-0.4	1.7
2	–2	0	1	–3	-	$C_{16}H_{12}O_5H^+$	285.0756	285.0758	-0.5	26.6
2	–2	0	1	–2	-	$C_{16}H_{14}O_5H^+$	287.0913	287.0914	-0.3	16.9
3	–3	–1	0	–3	-	$C_{18}H_{16}O_4H^+$	297.1121	297.1121	0.0	1.6
2	–2	0	2	–4	-	$C_{16}H_{12}O_6H^+$	301.0706	301.0707	-0.3	0.8
2	–2	0	2	–3	-	$C_{16}H_{14}O_6H^+$	303.0862	303.0863	-0.3	18.7
3	–3	–1	1	–2	-	$C_{18}H_{16}O_5H^+$	313.1070	313.1071	-0.3	2.6
3	–3	0	0	–4	-	$C_{24}H_{16}O_6H^+$	401.1017	401.1020	-0.7	0.8
3	–3	0	0	–3	-	$C_{24}H_{18}O_6H^+$	403.1174	403.1176	-0.5	1.6
3	–3	0	0	–2	-	$C_{24}H_{20}O_6H^+$	405.1330	405.1333	-0.6	4.3
3	–3	0	0	–1	-	$C_{24}H_{22}O_6H^+$	407.1487	407.1489	-0.5	2.4
4	–4	–1	0	–2	-	$C_{26}H_{22}O_6H^+$	431.1486	431.1489	-0.7	0.9
4	–4	–1	0	–1	-	$C_{26}H_{24}O_6H^+$	433.1642	433.1646	-0.9	1.5
4	–4	0	0	–4	-	$C_{32}H_{24}O_8H^+$	537.1541	537.1544	-0.6	1.0
4	–4	0	0	–3	-	$C_{32}H_{26}O_8H^+$	539.1696	539.1700	-0.7	1.8
4	–4	0	0	–2	-	$C_{32}H_{28}O_8H^+$	541.1852	541.1857	-0.9	1.1

Table S-16. Assignment of signals obtained by HRMS analysis of (P2+CA) at 220 °C for 5 min measured using an APCI-orbitrap-MS instrument in positive ion mode.

	structure assignment					mol.	*m/z*		rel. error	rel.
CA	CO_2	$C_6H_6O_2$	H_2O	H_2	other	formula	exp.	theo.	[ppm]	intensity
1	–1	0	1	–1	-	$C_8H_8O_3H^+$	153.0545	153.0546	-0.5	1.1
2	–2	–1	0	0	-	$C_{10}H_{10}O_2H^+$	163.0752	163.0754	-1.3	17.3
2	–2	–1	1	–2	-	$C_{10}H_8O_3H^+$	177.0545	177.0546	-0.8	2.6
2	–2	–1	1	–1	-	$C_{10}H_{10}O_3H^+$	179.0701	179.0703	-1.1	6.9
2	–2	0	–1	–1	-	$C_{16}H_{14}O_3H^+$	255.1014	255.1016	-0.8	1.0
2	–2	0	0	–1	$–CH_2$	$C_{15}H_{12}O_4H^+$	257.0806	257.0808	-0.9	3.1
2	–2	0	0	–1	$–CH_2$	$C_{15}H_{14}O_4H^+$	259.0962	259.0965	-1.2	1.1
2	–2	0	0	–3	-	$C_{16}H_{10}O_4H^+$	267.0650	267.0652	-0.9	1.9
2	–2	0	0	–2	-	$C_{16}H_{12}O_4H^+$	269.0806	269.0808	-0.7	20.2
2	–2	0	0	–1	-	$C_{16}H_{14}O_4H^+$	271.0961	271.0965	-1.3	100.0
2	–2	0	0	–1	-	$C_{16}H_{16}O_4H^+$	273.1118	273.1121	-0.9	2.3
2	–2	0	1	–4	-	$C_{16}H_{10}O_5H^+$	283.0599	283.0601	-0.7	2.0
2	–2	0	1	–3	-	$C_{16}H_{12}O_5H^+$	285.0755	285.0758	-0.9	33.5
3	–3	–1	0	0	$–CH_2$	$C_{17}H_{16}O_4H^+$	285.1120	285.1120	-0.1	1.5
2	–2	0	1	–2	-	$C_{16}H_{14}O_5H^+$	287.0912	287.0914	-0.8	10.2
3	–3	–1	0	–5	-	$C_{18}H_{12}O_4H^+$	293.0806	293.0808	-0.6	2.0
3	–3	–1	0	–4	-	$C_{18}H_{14}O_4H^+$	295.0963	295.0965	-0.7	1.7
3	–3	–1	0	–3	-	$C_{18}H_{16}O_4H^+$	297.1120	297.1121	-0.5	7.9
3	–3	–1	0	–2	-	$C_{18}H_{18}O_4H^+$	299.1276	299.1278	-0.8	11.2
2	–2	0	2	–4	-	$C_{16}H_{12}O_6H^+$	301.0704	301.0707	-1.0	1.0
2	–2	0	2	–3	-	$C_{16}H_{14}O_6H^+$	303.0861	303.0863	-0.7	10.1
3	–3	–1	1	–1	-	$C_{18}H_{14}O_5H^+$	311.0912	311.0914	-0.6	1.0
3	–3	–1	1	–2	-	$C_{18}H_{16}O_5H^+$	313.1068	313.1071	-1.0	3.0
3	–3	0	0	–5	-	$C_{24}H_{14}O_6H^+$	399.0859	399.0863	-1.0	2.4

3	–3	0	0	–4	-	$C_{24}H_{16}O_6H^+$	401.1016	401.1020	-1.1	4.1
3	–3	0	0	–3	-	$C_{24}H_{18}O_6H^+$	403.1172	403.1176	-1.0	12.8
3	–3	0	0	–2	-	$C_{24}H_{20}O_6H^+$	405.1328	405.1333	-1.1	41.4
3	–3	0	0	–1	-	$C_{24}H_{22}O_6H^+$	407.1484	407.1489	-1.2	17.8
3	–3	0	0	0	-	$C_{24}H_{24}O_6H^+$	409.1642	409.1646	-1.1	1.0
3	–3	0	1	–4	-	$C_{24}H_{18}O_7H^+$	419.1120	419.1125	-1.1	1.7
3	–3	0	1	–3	-	$C_{24}H_{20}O_7H^+$	421.1277	421.1282	-1.3	2.8
4	–4	–1	0	–3	-	$C_{26}H_{20}O_6H^+$	429.1327	429.1333	-1.4	1.4
4	–4	–1	0	–2	-	$C_{26}H_{22}O_6H^+$	431.1484	431.1489	-1.1	3.1
4	–4	–1	0	–1	-	$C_{26}H_{24}O_6H^+$	433.1640	433.1646	-1.4	5.0
4	–4	–1	0	0	-	$C_{26}H_{26}O_6H^+$	435.1797	435.1802	-1.1	3.7
4	–4	0	0	–4	-	$C_{32}H_{24}O_8H^+$	537.1538	537.1544	-1.1	1.4
4	–4	0	0	–3	-	$C_{32}H_{26}O_8H^+$	539.1694	539.1700	-1.1	3.7
4	–4	0	0	–2	-	$C_{32}H_{28}O_8H^+$	541.1851	541.1857	-1.2	6.4
4	–4	0	0	–1	-	$C_{32}H_{30}O_8H^+$	543.2007	543.2013	-1.1	4.8
5	–5	–1	0	–1	-	$C_{34}H_{32}O_8H^+$	569.2163	569.2163	0.1	1.2

Table S-17. Assignment of MS^2 and MS^3 spectra shown in Figure S-13.

spectrum	structure assignment								mol.	*m/z*		rel. error	rel.
	CA	CO_2	$C_7H_6O_2$	$C_6H_6O_2$	CO	CH_2	H_2O	H_2	formula	exp.	theo.	[ppm]	Int.
MS^3 at *m/z* 145	2	–2	–1	0	0	0	–2	0	$C_9H_6H^+$	115.0542	115.0542	0.0	1.4
	2	–2	–1	0	0	0	–2	1	$C_9H_8H^+$	117.0698	117.0699	-0.7	100.0
	2	–2	0	–1	0	0	–1	0	$C_{10}H_8OH^+$	145.0647	145.0648	-0.6	1.1
MS^2 at *m/z* 163	2	–2	–1	0	0	0	–2	1	$C_9H_8H^+$	117.0698	117.0699	-0.7	5.4
	2	–2	0	–1	0	0	–1	0	$C_{10}H_8OH^+$	145.0647	145.0648	-0.6	100.0
MS^2 at *m/z* 271	2	–2	–1	0	0	0	–2	0	$C_9H_6H^+$	115.0542	115.0542	0.0	3.8

	2	–2	0	–1	0	0	–1	–1	$C_{10}H_6OH^+$	143.0491	143.0491	0.0	20.3
	2	–2	0	–1	0	0	0	–1	$C_{10}H_8O_2H^+$	161.0596	161.0597	-0.6	100.0
	2	–2	0	0	0	–1	–2	1	$C_{15}H_{12}O_2H^+$	225.0911	225.0910	0.4	7.2
	2	–2	0	0	0	–1	–1	1	$C_{15}H_{14}O_3H^+$	243.1016	243.1016	0.1	38.6
	2	–2	0	0	0	0	–1	–1	$C_{16}H_{12}O_3H^+$	253.0859	253.0859	0.0	22.7

Table S-18. Assignment of MS^2 and MS^3 spectra shown in Figure S-14.

spectrum	structure assignment								mol.	*m/z*		rel. error	rel.
	CA	CO_2	$C_7H_6O_2$	$C_6H_6O_2$	CO	CH_2	H_2O	H_2	formula	exp.	theo.	[ppm]	Int.
MS^3 at *m/z* 257	2	–2	0	–1	0	–1	0	–2	$C_9H_6O_2H^+$	147.0441	147.0441	0.3	37.8
	2	–2	0	0	–2	–1	–1	–1	$C_{13}H_{10}OH^+$	183.0805	183.0804	0.3	3.5
	2	–2	0	0	–1	–1	–2	–1	$C_{14}H_8OH^+$	193.0649	193.0648	0.3	2.8
	2	–2	0	0	–1	–1	–1	–1	$C_{14}H_{10}O_2H^+$	211.0755	211.0754	0.6	100.0
	2	–2	0	0	0	–1	–2	–1	$C_{15}H_8O_2H^+$	221.0598	221.0597	0.6	14.1
	2	–2	0	0	–1	–1	0	–1	$C_{14}H_{12}O_3H^+$	229.0861	229.0859	0.7	3.7
	2	–2	0	0	0	–1	–1	–1	$C_{15}H_{10}O_3H^+$	239.0705	239.0703	0.8	56.5
	2	–2	0	0	0	–1	0	–1	$C_{15}H_{12}O_4H^+$	257.0810	257.0808	0.8	1.6
MS^2 at *m/z* 271	2	–2	–1	0	0	0	–2	0	$C_9H_6H^+$	115.0542	115.0542	0.0	3.8
	1	–1	0	0	0	–1	0	–1	$C_7H_6O_2H^+$	123.0441	123.0441	0.2	2.1
	2	–2	–1	0	0	0	–1	0	$C_9H_8OH^+$	133.0648	133.0648	0.1	0.4
	2	–2	0	–1	0	0	–1	–1	$C_{10}H_6OH^+$	143.0492	143.0491	0.5	15.0
	2	–2	0	–1	0	0	0	–1	$C_{10}H_8O_2H^+$	161.0597	161.0597	0.1	100.0
	2	–2	0	0	0	–1	–2	1	$C_{15}H_{10}OH^+$	207.0805	207.0804	0.4	0.7
	2	–2	0	0	0	–1	–2	1	$C_{15}H_{12}O_2H^+$	225.0912	225.0910	0.8	5.5
	2	–2	0	0	0	0	–2	–1	$C_{16}H_{10}O_2H^+$	235.0755	235.0754	0.7	2.3

	2	–2	0	0	0	–1	–1	1	$C_{15}H_{14}O_3H^+$	243.1017	243.1016	0.5	13.0
	2	–2	0	0	0	0	–1	–1	$C_{16}H_{12}O_3H^+$	253.0860	253.0859	0.3	20.7
	2	–2	0	0	0	0	0	–1	$C_{16}H_{14}O_4H^+$	271.0967	271.0965	0.7	0.2
MS^2 at *m/z* 285	2	–2	0	–1	0	–1	0	–2	$C_9H_6O_2H^+$	147.0441	147.0441	0.4	0.9
	2	–2	0	–1	0	0	1	–3	$C_{10}H_6O_3H^+$	175.0390	175.0390	0.4	2.6
	2	–2	0	0	–1	–1	–1	–1	$C_{14}H_{10}O_2H^+$	211.0755	211.0754	0.6	4.4
	2	–2	0	0	0	–1	–1	–1	$C_{15}H_{10}O_3H^+$	239.0705	239.0703	0.8	16.9
	2	–2	0	0	0	–1	0	–1	$C_{15}H_{12}O_4H^+$	257.0808	257.0808	-0.2	100.0
	2	–2	0	0	0	0	0	–3	$C_{16}H_{10}O_4H^+$	267.0653	267.0652	0.5	9.9
	2	–2	0	0	0	0	1	–3	$C_{16}H_{12}O_5H^+$	285.0760	285.0758	0.7	1.4
MS^3 at *m/z* 295	3	–3	0	–1	–1	0	–1	–2	$C_{17}H_{12}O_2H^+$	249.0911	249.0910	0.5	10.9
	3	–3	0	–1	–1	0	0	–2	$C_{17}H_{14}O_3H^+$	267.1018	267.1016	0.7	2.3
	3	–3	0	–1	0	0	–1	–2	$C_{18}H_{12}O_3H^+$	277.0860	277.0859	0.3	100.0
MS^2 at *m/z* 405	1	–1	0	0	0	–1	0	–1	$C_7H_6O_2H^+$	123.0441	123.0441	0.2	2.1
	1	–1	0	0	0	0	0	0	$C_8H_8O_2H^+$	137.0597	137.0597	0.1	1.4
	3	–3	0	–1	0	–1	–2	0	$C_{17}H_{12}O_2H^+$	249.0911	249.0910	0.5	5.0
	2	–2	0	0	0	0	0	–2	$C_{16}H_{12}O_4H^+$	269.0810	269.0808	0.7	6.8
	3	–3	0	–1	0	0	–1	–2	$C_{18}H_{12}O_3H^+$	277.0860	277.0859	0.4	44.6
	3	–3	0	–1	0	–1	0	–1	$C_{17}H_{14}O_4H^+$	283.0967	283.0965	0.8	4.6
	3	–3	0	–1	0	0	0	–2	$C_{18}H_{14}O_4H^+$	295.0965	295.0965	0.1	100.0
	4	–4	0	0	–1	0	–1	–2	$C_{23}H_{18}O_4H^+$	359.1281	359.1278	0.9	3.0
	4	–4	0	0	0	0	–2	–2	$C_{24}H_{16}O_4H^+$	369.1125	369.1121	0.9	3.6
	4	–4	0	0	0	0	–1	–2	$C_{24}H_{18}O_5H^+$	387.1230	387.1227	0.8	18.5
MS^2 at *m/z* 433	1	–1	0	0	0	0	0	0	$C_8H_8O_2H^+$	137.0597	137.0597	0.0	4.3
	2	–2	0	–1	0	0	–1	–1	$C_{10}H_6OH^+$	143.0492	143.0491	0.4	1.8
	3	–3	1	–2	0	0	–1	0	$C_{10}H_8OH^+$	145.0648	145.0648	0.2	15.1

	2	–2	0	–1	0	0	0	–1	$C_{10}H_8O_2H^+$	161.0598	161.0597	0.4	14.6
	2	–2	0	0	0	–1	0	–1	$C_{15}H_{12}O_4H^+$	257.0810	257.0808	0.6	0.9
	2	–2	0	0	0	0	0	–1	$C_{16}H_{14}O_4H^+$	271.0967	271.0965	0.6	24.2
	3	–3	0	–1	0	0	–1	–1	$C_{18}H_{14}O_3H^+$	279.1017	279.1016	0.6	44.6
	3	–3	0	–1	0	–1	0	–2	$C_{17}H_{12}O_4H^+$	281.0811	281.0808	1.1	0.8
	3	–3	0	–1	0	–1	0	–1	$C_{17}H_{14}O_4H^+$	283.0967	283.0965	0.8	10.9
	3	–3	0	–1	0	0	0	–2	$C_{18}H_{14}O_4H^+$	295.0967	295.0965	0.8	1.6
	3	–3	0	–1	0	0	0	–1	$C_{18}H_{16}O_4H^+$	297.1123	297.1121	0.5	81.0
	4	–4	0	–2	0	0	–1	–1	$C_{20}H_{16}O_3H^+$	305.1175	305.1172	0.8	12.8
	4	–4	0	–2	0	–1	0	0	$C_{19}H_{18}O_4H^+$	311.1281	311.1278	0.9	2.1
	4	–4	0	–2	0	–1	1	–3	$C_{19}H_{14}O_5H^+$	323.0918	323.0914	1.3	0.8
	4	–4	0	–2	0	0	0	–1	$C_{20}H_{18}O_3H^+$	323.1280	323.1278	0.7	100.0
	4	–4	0	–1	0	0	–1	–1	$C_{26}H_{22}O_5H^+$	415.1541	415.1540	0.3	3.5
	4	–4	0	–1	0	0	–1	–1	$C_{26}H_{24}O_6H^+$	433.1648	433.1646	0.5	0.6

Table S-19. Assignment of MS^2 and MS^3 spectra shown in Figure S-15.

spectrum	structure assignment								mol.	*m/z*		rel. error	rel.
	CA	CO_2	$C_7H_6O_2$	$C_6H_6O_2$	CO	CH_2	H_2O	H_2	formula	exp.	theo.	[ppm]	Int.
MS^2 at *m/z* 299	2	–2	–1	0	0	0	–2	1	$C_9H_8H^+$	117.0700	117.0699	0.7	0.8
	1	–1	0	0	0	–1	0	–1	$C_7H_6O_2H^+$	123.0441	123.0441	0.5	1.8
	1	–1	0	0	0	0	0	0	$C_8H_8O_2H^+$	137.0598	137.0597	0.5	3.9
	2	–2	0	–1	0	0	–1	–1	$C_{10}H_6OH^+$	143.0492	143.0491	0.9	1.7
	2	–2	0	–1	0	0	–1	–2	$C_{10}H_8OH^+$	145.0649	145.0648	0.6	9.8
	2	–2	0	–1	0	0	0	–1	$C_{10}H_8O_2H^+$	161.0598	161.0597	0.9	16.3
	2	–2	0	–1	0	0	0	–1	$C_{10}H_{10}O_2H^+$	163.0754	163.0754	0.3	52.7
	3	–3	0	–2	0	0	1	–3	$C_{12}H_{10}OH^+$	171.0806	171.0804	0.6	4.7

	3	–3	0	–2	0	–1	0	–1	$C_{11}H_{10}O_2H^+$	175.0755	175.0754	0.7	2.4
	3	–3	0	–2	0	0	0	–3	$C_{12}H_{12}O_2H^+$	189.0909	189.0910	-0.4	100.0
	3	–3	0	–1	0	–1	–2	1	$C_{17}H_{16}O_2H^+$	253.1225	253.1223	0.9	1.8
	2	–2	0	0	0	–1	0	–1	$C_{15}H_{12}O_4H^+$	257.0810	257.0808	0.8	15.5
	3	–3	0	–1	0	0	–2	–1	$C_{18}H_{14}O_2H^+$	263.1069	263.1067	1.0	1.0
	2	–2	0	0	0	–1	1	–4	$C_{15}H_{14}O_5H^+$	271.0604	271.0601	1.0	1.1
	2	–2	0	0	0	0	0	–1	$C_{16}H_{14}O_4H^+$	271.0968	271.0965	1.0	1.2
	3	–3	0	–1	0	–1	0	–3	$C_{17}H_{12}O_4H^+$	281.0811	281.0808	1.1	0.9
	3	–3	0	–1	0	0	–1	–1	$C_{18}H_{16}O_3H^+$	281.1175	281.1172	1.0	7.2
MS[3] at *m/z* 323	1	–1	0	0	0	–1	0	–1	$C_7H_6O_2H^+$	123.0441	123.0441	0.1	1.2
	2	–2	0	–1	0	–1	0	–1	$C_9H_8O_2H^+$	149.0597	149.0591	4.0	1.4
	2	–2	0	–1	0	0	0	–1	$C_{10}H_8O_2H^+$	161.0598	161.0597	0.5	5.6
	4	–4	0	–2	0	–1	–2	–2	$C_{19}H_{16}O_2H^+$	277.1225	277.1223	0.7	8.0
	3	–3	0	–1	0	–1	0	–3	$C_{17}H_{12}O_4H^+$	281.0811	281.0808	0.9	6.5
	4	–4	0	–2	0	0	–2	–1	$C_{20}H_{14}O_2H^+$	287.1069	287.1067	0.8	8.0
	3	–3	0	–1	0	0	0	–2	$C_{18}H_{14}O_4H^+$	295.0969	295.0965	1.2	1.4
	4	–4	0	–2	0	0	–1	–1	$C_{20}H_{16}O_3H^+$	305.1176	305.1172	1.4	100.0

Table S-20. Assignment of MS^2 and MS^3 spectra shown in Figure S-16.

**spectrum**	**structure assignment**								**mol.**	***m/z***		**rel. Error**	**rel.**
	**CA**	**CO_2**	**$C_7H_6O_2$**	**$C_6H_6O_2$**	**CO**	**CH_2**	**H_2O**	**H_2**	**formula**	**exp.**	**theo.**	**[ppm]**	**Int.**
MS[2] at *m/z* 271	2	–2	–1	0	0	0	–2	0	$C_9H_6H^+$	115.0542	115.0542	0.0	3.7
	1	–1	0	0	0	–1	0	0	$C_7H_6O_2H^+$	123.0440	123.0441	-0.5	2.6
	2	–2	0	–1	0	–1	–1	0	$C_9H_8OH^+$	133.0648	133.0648	0.1	0.6
	2	–2	0	–1	0	0	–1	–1	$C_{10}H_6OH^+$	143.0491	143.0491	0.0	20.4

	2	–2	0	–1	0	0	0	–1	$C_{10}H_8O_2H^+$	161.0596	161.0597	-0.6	100.0
	2	–2	0	0	–1	0	–2	–1	$C_{15}H_{10}OH^+$	207.0804	207.0804	-0.2	1.2
	2	–2	0	0	0	–1	–2	1	$C_{15}H_{12}O_2H^+$	225.0911	225.0910	0.4	7.2
	2	–2	0	0	0	0	–2	–1	$C_{16}H_{10}O_2H^+$	235.0754	235.0754	0.2	3.3
	2	–2	0	0	0	–1	–1	1	$C_{15}H_{14}O_3H^+$	243.1016	243.1016	0.1	27.0
	2	–2	0	0	0	0	–1	–1	$C_{16}H_{12}O_3H^+$	253.0858	253.0859	-0.4	22.5
MS^2 at *m/z* 407	1	–1	0	0	0	0	0	0	$C_8H_8O_2H^+$	137.0597	137.0597	-0.1	3.9
	2	–2	0	–1	0	–1	0	–1	$C_9H_6O_2H^+$	147.0441	147.0441	0.3	0.5
	2	–2	0	0	0	–1	–1	–1	$C_{15}H_{10}O_3H^+$	239.0703	239.0703	0.1	2.4
	2	–2	0	0	0	0	–1	–1	$C_{16}H_{12}O_3H^+$	253.0860	253.0859	0.3	1.1
	2	–2	0	0	0	–1	0	–1	$C_{15}H_{12}O_4H^+$	257.0808	257.0808	-0.2	100.0
	2	–2	0	0	0	0	0	–2	$C_{16}H_{12}O_4H^+$	269.0810	269.0808	0.6	1.6
	2	–2	0	0	0	0	0	–1	$C_{16}H_{14}O_4H^+$	271.0966	271.0965	0.4	16.9
	3	–3	0	–1	0	0	–1	–1	$C_{18}H_{14}O_3H^+$	279.1017	279.1016	0.5	4.6
	3	–3	0	–1	0	–1	0	0	$C_{17}H_{16}O_4H^+$	285.1123	285.1121	0.6	1.5
	3	–3	0	–1	0	0	0	–1	$C_{18}H_{16}O_4H^+$	297.1122	297.1121	0.2	49.8
	3	–3	0	0	0	–2	–2	1	$C_{22}H_{18}O_4H^+$	347.1281	347.1278	0.9	0.8
	3	–3	0	0	0	–1	–2	1	$C_{23}H_{20}O_4H^+$	361.1437	361.1434	0.7	5.9
	3	–3	0	0	0	–3	0	–1	$C_{21}H_{16}O_6H^+$	365.1022	365.1020	0.7	4.2
	3	–3	0	0	0	0	–2	1	$C_{24}H_{18}O_4H^+$	371.1281	371.1278	0.8	2.3
	3	–3	0	0	0	0	–1	–1	$C_{24}H_{20}O_5H^+$	389.1386	389.1384	0.6	85.8

Table S-21. Assignment of MS² and MS³ spectra shown in Figure S-17.

spectrum	structure assignment								mol. formula	*m/z*		rel. error [ppm]	rel. Int.
	CA	CO_2	$C_7H_6O_2$	$C_6H_6O_2$	CO	CH_2	H_2O	H_2		exp.	theo.		
MS² at *m/z* 299	2	–2	–1	0	0	0	–2	1	$C_9H_8H^+$	117.0699	117.0699	0.1	0.9
	1	–1	0	0	0	–1	0	0	$C_7H_7O_2H^+$	123.0441	123.0441	0.0	0.9
	1	–1	0	0	0	0	0	0	$C_8H_9O_2H^+$	137.0597	137.0597	-0.1	4.1
	2	–2	0	–1	0	0	–1	–2	$C_{10}H_6OH^+$	143.0491	143.0491	0.0	0.7
	2	–2	0	–1	0	0	–1	–1	$C_{10}H_8OH^+$	145.0648	145.0648	0.0	12.7
	2	–2	0	–1	0	0	0	–1	$C_{10}H_8O_2H^+$	161.0598	161.0597	0.4	9.3
	2	–2	0	–1	0	0	0	0	$C_{10}H_{10}O_2H^+$	163.0753	163.0754	-0.1	60.1
	3	–3	0	–2	0	0	–1	0	$C_{12}H_{10}OH^+$	171.0805	171.0804	0.2	3.5
	3	–3	0	–2	0	0	0	0	$C_{12}H_{12}O_2H^+$	189.0908	189.0908	0.1	100.0
	2	–2	0	0	0	0	–1	–1	$C_{16}H_{12}O_3H^+$	253.0860	253.0859	0.2	0.6
	2	–2	0	0	0	–1	0	–1	$C_{15}H_{12}O_4H^+$	257.0809	257.0808	0.2	10.2
	2	–2	0	0	0	0	0	–1	$C_{16}H_{14}O_4H^+$	271.0966	271.0965	0.4	1.0
	3	–3	0	–1	0	–1	–1	2	$C_{17}H_{18}O_3H^+$	271.1329	271.1329	0.3	0.5
	3	–3	0	–1	0	0	–1	0	$C_{18}H_{16}O_3H^+$	281.1173	281.1172	0.4	4.0
MS³ at *m/z* 431	2	–2	0	0	0	–3	0	0	$C_{13}H_{10}O_4H^+$	231.0653	231.0652	0.4	1.4
	2	–2	0	0	0	0	0	–2	$C_{16}H_{12}O_4H^+$	269.0807	269.0808	-0.3	4.8
	3	–3	0	–1	0	0	–1	–2	$C_{18}H_{12}O_3H^+$	277.0858	277.0859	-0.3	2.6
	3	–3	0	–1	0	0	0	–2	$C_{18}H_{12}O_4H^+$	293.0807	293.0807	0.1	1.5
	3	–3	0	–1	0	0	0	–2	$C_{18}H_{14}O_4H^+$	295.0964	295.0965	-0.4	41.0
	4	–4	0	–2	0	0	–1	–2	$C_{20}H_{14}O_3H^+$	303.1015	303.1015	0.0	33.1
	4	–4	0	–2	0	–1	0	–2	$C_{19}H_{14}O_4H^+$	307.0966	307.0965	0.4	2.2
	4	–4	0	–2	0	–1	0	–1	$C_{19}H_{16}O_4H^+$	309.1121	309.1121	-0.2	6.3
	4	–4	0	–2	0	0	0	–2	$C_{20}H_{16}O_4H^+$	321.1120	321.1121	-0.4	91.7

	4	–4	0	–1	0	–1	–2	0	$C_{25}H_{20}O_4H^+$	385.1431	385.1434	-0.8	2.6
	3	–3	0	0	0	–1	0	–3	$C_{23}H_{16}O_6H^+$	389.1019	389.1020	-0.3	2.6
	4	–4	0	–1	0	0	–2	–2	$C_{26}H_{18}O_4H^+$	395.1272	395.1278	-1.5	1.8
	4	–4	0	–1	0	0	–1	–2	$C_{26}H_{20}O_5H^+$	413.1380	413.1381	-0.3	34.9
	4	–4	0	–1	0	0	0	–2	$C_{26}H_{22}O_6H^+$	431.1485	431.1489	-0.9	100.0
	2	–2	0	0	0	0	0	–2	$C_{16}H_{12}O_4H^+$	269.0808	269.0808	0.0	2.5
	2	–2	0	0	0	0	0	–1	$C_{16}H_{14}O_4H^+$	271.0964	271.0965	-0.3	2.6
	3	–3	0	–1	0	0	–1	–2	$C_{18}H_{12}O_3H^+$	277.0859	277.0859	-0.2	2.2
	3	–3	0	–1	0	–1	0	–2	$C_{17}H_{12}O_4H^+$	281.0808	281.0808	-0.3	2.1
	3	–3	0	–1	0	0	0	–2	$C_{18}H_{14}O_4H^+$	295.0964	295.0965	-0.2	15.6
	3	–3	0	–1	0	0	0	–1	$C_{18}H_{16}O_4H^+$	297.1121	297.1121	-0.1	2.3
MS^2 at	4	–4	0	–2	0	0	0	–2	$C_{20}H_{16}O_4H^+$	321.1121	321.1121	-0.2	3.3
m/z 541	3	–3	0	0	0	–1	0	–2	$C_{23}H_{18}O_6H^+$	391.1175	391.1176	-0.2	16.8
	3	–3	0	0	0	0	0	–3	$C_{24}H_{18}O_6H^+$	403.1174	403.1176	-0.6	2.3
	3	–3	0	0	0	0	0	–2	$C_{24}H_{20}O_6H^+$	405.1331	405.1333	-0.5	44.3
	4	–4	0	–1	0	0	–1	–2	$C_{26}H_{20}O_5H^+$	413.1381	413.1384	-0.5	1.4
	4	–4	0	–1	0	0	0	–2	$C_{26}H_{22}O_6H^+$	431.1485	431.1489	-0.8	100.0
	4	–4	0	0	0	0	–1	–2	$C_{32}H_{26}O_7H^+$	523.1747	523.1751	-0.7	3.5
	4	–4	0	0	0	0	0	–2	$C_{32}H_{28}O_8H^+$	541.1854	541.1857	-0.5	1.6

Table S-22. Assignment of MS² spectra shown in Figure S-18.

spectrum	structure assignment								mol.	*m/z*		rel. error	rel.
	CA	CO_2	$C_7H_6O_2$	$C_6H_6O_2$	CO	CH_2	H_2O	H_2	formula	exp.	theo.	[ppm]	Int.
MS² at *m/z* 271	2	–2	–1	0	0	0	–2	0	$C_9H_6H^+$	115.0542	115.0542	-0.1	3.1
	1	–1	0	0	0	–1	0	0	$C_7H_6O_2H^+$	123.0440	123.0441	-0.6	2.6
	2	–2	0	–1	0	–1	–1	0	$C_9H_8OH^+$	133.0647	133.0648	-0.5	0.3
	2	–2	0	–1	0	0	–1	–1	$C_{10}H_6OH^+$	143.0491	143.0491	-0.2	17.9
	2	–2	0	–1	0	0	0	–1	$C_{10}H_8O_2H^+$	161.0596	161.0597	-0.6	100.0
	2	–2	0	0	–1	0	–2	–1	$C_{15}H_{10}OH^+$	207.0804	207.0804	-0.3	0.9
	2	–2	0	0	0	–1	–2	1	$C_{15}H_{12}O_2H^+$	225.0910	225.0910	0.1	6.5
	2	–2	0	0	0	0	–2	–1	$C_{16}H_{10}O_2H^+$	235.0754	235.0754	0.1	7.2
	2	–2	0	0	0	–1	–1	1	$C_{15}H_{14}O_3H^+$	243.1015	243.1016	-0.3	3.6
	2	–2	0	0	0	0	–1	–1	$C_{16}H_{12}O_3H^+$	253.0857	253.0859	-0.6	50.0
MS² at *m/z* 297	1	–1	0	0	0	–1	0	0	$C_7H_6O_2H^+$	123.0440	123.0441	-0.8	100.0
	1	–1	0	0	0	0	0	0	$C_8H_8O_2H^+$	137.0597	137.0597	0.3	4.8
	3	–3	–1	0	0	0	–2	0	$C_{17}H_{14}O_2H^+$	251.1068	251.1067	0.4	37.4
	2	–2	0	0	0	–1	0	–2	$C_{15}H_{10}O_4H^+$	255.0654	255.0652	0.7	3.1
	3	–3	0	–1	0	0	–2	–1	$C_{18}H_{12}O_2H^+$	261.0912	261.0910	0.8	8.5
	3	–3	0	–1	0	0	–1	–1	$C_{18}H_{14}O_3H^+$	279.1016	279.1016	0.1	82.1
MS² at *m/z* 435	1	–1	0	0	0	0	0	0	$C_8H_8O_2H^+$	137.0596	137.0597	-0.9	31.8
	2	–2	0	–1	0	0	–1	0	$C_{10}H_8OH^+$	145.0647	145.0648	-0.9	9.3
	2	–2	0	–1	0	–1	0	0	$C_9H_8O_2H^+$	149.0596	149.0597	-0.7	11.2
	2	–2	–1	0	0	0	0	0	$C_9H_{10}O_2H^+$	151.0753	151.0754	-0.6	2.2
	2	–2	0	–1	0	0	0	0	$C_{10}H_{10}O_2H^+$	163.0752	163.0754	-0.8	100.0

	2	–2	0	–1	0	0	0	1	$C_{10}H_{12}O_2H^+$	165.0909	165.0910	-0.8	1.5
	3	–3	–1	–1	0	0	0	–1	$C_{11}H_{10}O_2H^+$	175.0752	175.0754	-0.7	10.0
	3	–3	0	–2	0	0	0	0	$C_{12}H_{12}O_2H^+$	189.0909	189.0908	0.4	9.6
	2	–2	0	0	0	–1	0	–1	$C_{15}H_{12}O_4H^+$	257.0808	257.0808	-0.3	9.4
	2	–2	0	0	0	–1	0	–1	$C_{15}H_{14}O_4H^+$	259.0964	259.0965	-0.5	1.2
	2	–2	0	0	0	0	0	–1	$C_{16}H_{14}O_4H^+$	271.0964	271.0965	-0.3	16.3
	2	–2	0	0	0	0	0	–1	$C_{16}H_{16}O_4H^+$	273.1121	273.1121	0.1	0.8
	3	–3	–1	0	0	0	0	–2	$C_{17}H_{14}O_4H^+$	283.0965	283.0965	-0.1	19.5
	3	–3	–1	0	0	0	0	–1	$C_{17}H_{16}O_4H^+$	285.1121	285.1120	0.3	16.1
	3	–3	0	–1	0	0	0	–4	$C_{18}H_{14}O_4H^+$	295.0965	295.0965	0.1	2.9
	3	–3	0	–1	0	0	0	–3	$C_{18}H_{16}O_4H^+$	297.1121	297.1121	-0.1	53.4
	3	–3	0	–1	0	0	0	–2	$C_{18}H_{18}O_4H^+$	299.1278	299.1278	0.0	18.9
	4	–4	0	–2	0	0	–1	0	$C_{20}H_{18}O_3H^+$	307.1327	307.1329	-0.6	0.9
	4	–4	0	–2	0	–1	0	0	$C_{19}H_{18}O_4H^+$	311.1278	311.1278	0.0	1.3
	4	–4	0	–2	0	–1	0	1	$C_{19}H_{20}O_4H^+$	313.1434	313.1434	-0.2	2.7
	4	–4	0	–2	0	0	1	–3	$C_{19}H_{16}O_5H^+$	325.1070	325.1071	-0.1	55.7
	4	–4	0	–2	0	0	0	0	$C_{20}H_{20}O_4H^+$	325.1434	325.1434	-0.1	92.4
	4	–4	0	–1	0	–1	–2	2	$C_{25}H_{24}O_4H^+$	389.1747	389.1747	-0.2	3.8
	4	–4	0	–1	0	0	–2	0	$C_{26}H_{22}O_4H^+$	399.1590	399.1591	-0.3	3.5
	4	–4	0	–1	0	–2	1	–4	$C_{24}H_{16}O_7H^+$	417.0967	417.0967	0.0	1.5
	4	–4	0	–1	0	0	–1	0	$C_{26}H_{24}O_5H^+$	417.1695	417.1697	-0.3	22.6

Table S-23. Assignment of MS[2] spectrum shown in Figure S-19.

spectrum	structure assignment								mol.	*m/z*		rel. error	rel.
	CA	CO_2	$C_7H_6O_2$	$C_6H_6O_2$	CO	CH_2	H_2O	H_2	formula	exp.	theo.	[ppm]	Int.
MS[2] at *m/z* 539	2	–2	0	0	0	0	0	–2	$C_{16}H_{12}O_4H^+$	269.0811	269.0808	1.4	2.4
	2	–2	0	0	0	0	0	–1	$C_{16}H_{14}O_4H^+$	271.0967	271.0965	0.9	0.9
	3	–3	0	–1	0	0	0	–3	$C_{18}H_{12}O_4H^+$	293.0812	293.0808	1.3	11.3
	3	–3	0	–1	0	0	0	–2	$C_{18}H_{14}O_4H^+$	295.0968	295.0965	1.0	2.9
	3	–3	0	0	0	–1	0	–3	$C_{23}H_{16}O_6H^+$	389.1023	389.1020	0.9	10.0
	3	–3	0	0	0	0	0	–4	$C_{24}H_{16}O_6H^+$	401.1023	401.1020	0.9	4.2
	3	–3	0	0	0	0	0	–3	$C_{24}H_{18}O_6H^+$	403.1179	403.1176	0.8	27.3
	4	–4	0	–1	0	0	–1	–3	$C_{26}H_{18}O_5H^+$	411.1230	411.1227	0.7	7.2
	4	–4	0	–1	0	0	0	–3	$C_{26}H_{20}O_6H^+$	429.1335	429.1333	0.5	100.0
	4	–4	0	0	0	–1	–2	–1	$C_{31}H_{24}O_6H^+$	493.1647	493.1646	0.3	1.8
	4	–4	0	0	0	–3	0	–3	$C_{29}H_{20}O_8H^+$	497.1234	497.1231	0.6	3.3
	4	–4	0	0	0	–1	0	–5	$C_{31}H_{20}O_8H^+$	521.1235	521.1231	0.7	1.2
	4	–4	0	0	0	0	–1	–3	$C_{32}H_{24}O_7H^+$	521.1598	521.1595	0.5	16.4
	4	–4	0	0	0	0	0	–3	$C_{32}H_{26}O_8H^+$	539.1703	539.1700	0.5	13.4
